# Vertical Self‐Rectifying Memristive Arrays for Page‐Wise Parallel Logic and Arithmetic Processing

**DOI:** 10.1002/adma.202514099

**Published:** 2025-11-24

**Authors:** Kunhee Son, Jea Min Cho, Dong Hoon Shin, Yeong Rok Kim, Néstor Ghenzi, Sunwoo Cheong, Byeong Su Kim, Jung Kyu Lee, Sungho Kim, Wonho Choi, Soo Hyung Lee, Janguk Han, Cheol Seong Hwang

**Affiliations:** ^1^ Department of Materials Science and Engineering and Inter‐university Semiconductor Research Center, College of Engineering Seoul National University Seoul 08826 Republic of Korea; ^2^ Universidad de Avellaneda UNDAV and Consejo Nacional de Investigaciones Científicas y Técnicas (CONICET) Mario Bravo 1460 Avellaneda Buenos Aires 1872 Argentina

**Keywords:** arithmetic logic unit, logic‐in‐memory, memristive logic, self‐rectifying memristor, vertical memristor

## Abstract

Logic‐in‐memory (LIM) architectures are explored to address the data transfer bottleneck of conventional von Neumann architectures by integrating computation directly within memory arrays. Among various candidates, memristor‐based LIM systems have gained significant attention due to their non‐volatile switching behavior and compatibility with dense integration. In this work, a page‐wise LIM architecture is implemented using a 3D vertical resistive random‐access memory array composed of self‐rectifying Pt–Ta_2_O_5_–Al:HfO_2_–TiN memristors. Two logic primitives—1 M and 2 M logic—are employed to perform intra‐page and inter‐page operations, respectively, enabling core Boolean functions to be executed entirely within the array through resistive state transitions. Based on these logic operations, a memristive arithmetic logic unit (mALU) is designed to perform essential arithmetic functions, including addition, subtraction, increment, and decrement. Owing to the vertical structure of vertical resistive random‐access memory, a 2‐bit full adder is implemented with a footprint of only three cells and completed in 12 steps. The proposed intra‐and inter‐page operations, along with complete mALU functionality, are experimentally demonstrated with high reproducibility. Combined with significantly reduced spatiotemporal cost, these results highlight the promise of this architecture for scalable and energy‐efficient in‐memory computing.

## Graphical abstract

This study proposes a page‐wise logic‐in‐memory architecture realized in a 3D vertical resistvie random‐access memory array of self‐rectifying memristors. By introducing intra‐ and inter‐page logic primitives, the system enables Boolean and arithmetic operations to be executed directly within the memory. A memristive arithmetic logic unit is experimentally demonstrated, including a compact 2‐bit full‐adder, validating high reproducibility and reduced spatiotemporal cost. These results establish a scalable and energy‐efficient pathway toward in‐memory computing with vertical memristive arrays.

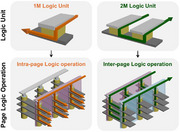

## 1 Introduction

The increasing demand for data‐intensive and low‐power computing has revealed the inherent limitations of the conventional von Neumann architecture, which physically separates processing and memory units.^[^ ^1^ ^]^ This separation leads to the so‐called von Neumann bottleneck, where frequent data shuttling between logic and memory imposes severe penalties on both energy efficiency and computational throughput. To address this challenge, logic‐in‐memory (LIM) paradigms have emerged as promising alternatives, aiming to integrate computation and storage within a unified physical substrate to reduce data movement and enhance efficiency.^[^ ^2^, ^3^, ^4^, ^5^ ^]^

Among various candidate technologies for enabling LIM architectures, memristors have received significant attention due to their ability to function simultaneously as memory and logic elements.^[^ ^6^, ^7^, ^8^, ^9^, ^10^ ^]^ Their non‐volatile resistance states, state tunability, low power consumption, and compatibility with silicon back‐end‐of‐line processing make them especially well‐suited for dense, energy‐efficient computing‐in‐memory systems.^[^ ^11^, ^12^, ^13^, ^14^, ^15^, ^16^, ^17^, ^18^, ^19^ ^]^ A wide range of memristor‐based logic operations, including stateful and sequential logic gating, thresholding functions, and resistance‐coded arithmetic operations, have been demonstrated using 2D crossbar arrays, validating their functional versatility at both the device and array levels.^[^ ^20^, ^21^, ^22^, ^23^ ^]^

However, these 2D crossbar arrays face inherent limitations regarding the scalability, interconnect complexity, and limited parallelism. These limitations are becoming increasingly critical when implementing practical applications that require extensive cascading and massive parallelism. 3D integration of the memristor array is a viable option to address these issues. The semiconductor industry has already adopted a 3D stacking strategy to mitigate scaling issues in memory, as evidenced by the commercial adoption of vertical NAND flash, which stacks charge trap flash memory layers. In line with this trend, memristor technologies are also evolving toward 3D integration, resulting in the development of vertical resistive random‐access memory (V‐RRAM) architectures with higher integration density and smaller footprint.^[^ ^24^, ^25^, ^26^ ^]^ Still, the sneak current issue must be addressed when the V‐RRAM architecture is adopted for the LIM applications. Integrating a cell selection transistor or selector, such as diodes, may not be a viable option for implementing the crucial merits of memristors in 3D crossbar arrays. In this regard, self‐rectifying memristors (SRMs) stand out as a particularly effective solution for 3D integration due to their asymmetric current‐voltage (I–V) characteristics, which inherently suppress reverse current flow and eliminate the need for transistors or selectors.^[^ ^27^ ^]^ This selector‐free operation not only mitigates sneak‐path currents—a major challenge in high‐density crossbar arrays—but also simplifies fabrication and enhances scalability, making SRMs highly advantageous for vertical LIM implementations. It is noted that the cell disturbance caused by the sneak current is a less severe problem when passive crossbar arrays are used for vector‐matrix multiplication in neural network applications, where the simultaneous input of voltages to multiple word lines (WLs) mitigates the issue. However, LIM needs more careful suppression of this issue as the inputs are more independently controlled.

Beyond their physical advantages, SRMs exhibit uniform and stable resistive switching behavior,^[^ ^27^, ^28^ ^]^ making them suitable for reliable LIM execution. Building on these properties, previous studies feasibly exploited SRMs to implement basic Boolean logic using SRM‐based V‐RRAM arrays.^[^ ^29^, ^30^ ^]^ However, these implementations primarily relied on line‐wise logic operations, where inputs and outputs are distributed across WLs or bit lines (BLs). This configuration inherently limits the degree of achievable parallelism and poses challenges for large and complex computing workloads. Crucially, the vertical dimension of the V‐RRAM array has not been fully exploited for parallel computation in these architectures.

**Figure 1:**
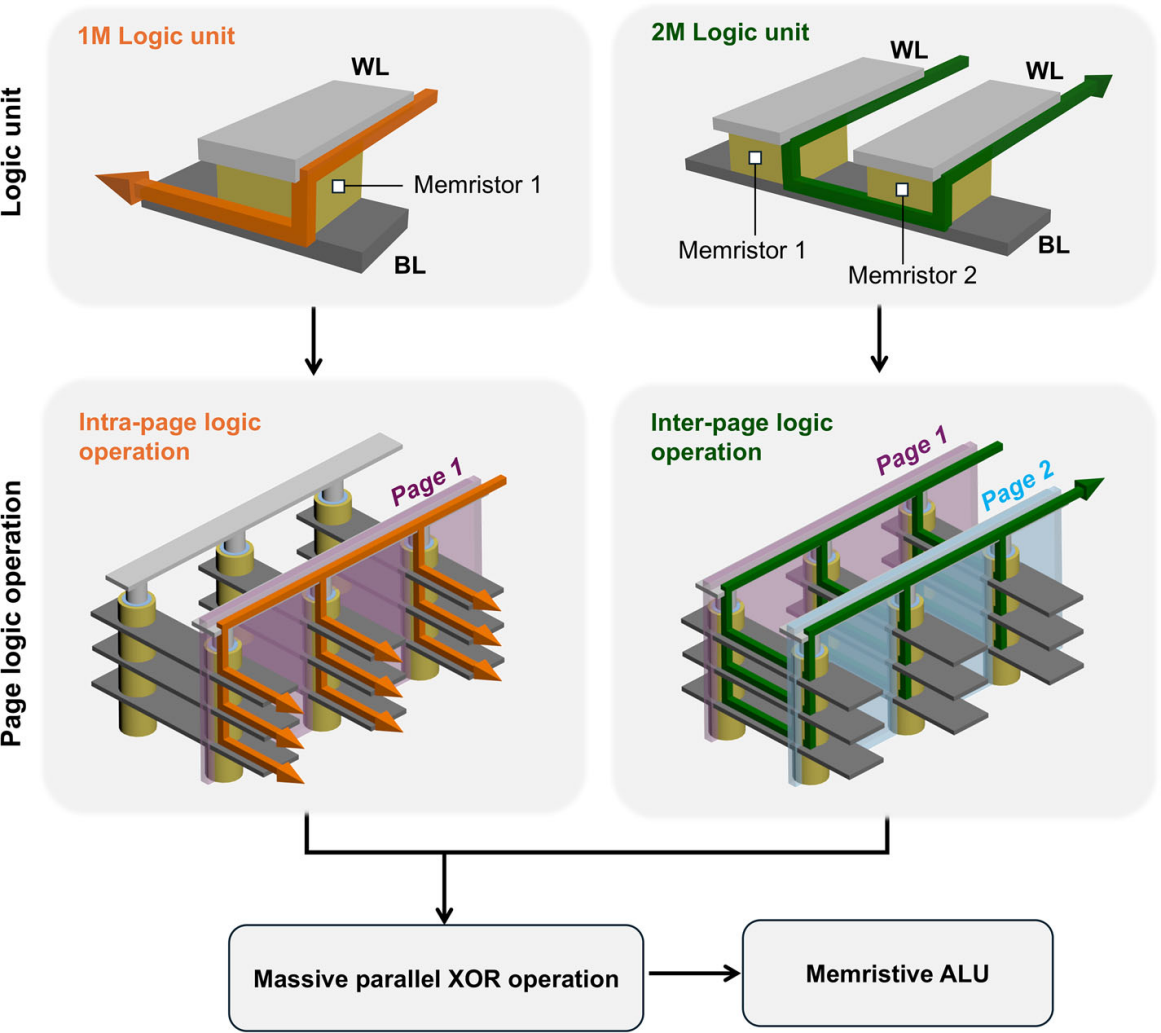
Conceptual schematic diagram of the proposed page‐wise LIM architecture implemented using vertically integrated self‐rectifying memristive arrays. The combination of intra‐page and inter‐page logic operations enables parallel logic computation at the page level.

To overcome these limitations, this work proposes a new computing method that enables page‐wise parallel logic operations within a V‐RRAM array. A page refers to a vertically aligned group of devices sharing the same WL, serving as the fundamental unit of computation. **Figure** **1** illustrates the implemented memristor‐based logic units, confirming the feasibility of page‐wise logic operations within the V‐RRAM structure. Two types of fundamental logic operations are employed: 1 M logic and 2 M logic. The 1 M logic unit utilizes a single memristor per operation, where both the resistance state of the memristor and the applied voltage serve as logic inputs, and the final resistance state of the memristor represents the logic output. Therefore, it belongs to the family of sequential logic. In contrast, the 2 M logic unit involves two memristors per operation, with the resistance states of both devices acting as logic inputs. Upon applying a specific operation voltage, the final resistance state of one memristor determines the logic output. Therefore, it belongs to the family of stateful logic. When implemented within the V‐RRAM array, 1 M and 2 M logic operations are naturally extended to intra‐ and inter‐page operations, respectively. Specifically, utilizing 1 M logic enables intra‐page operations, wherein both input and output are confined to a single page within the array. In contrast, 2 M logic facilitates inter‐page operations by integrating inputs distributed across two distinct pages. Therefore, this work also presents a method to overcome the inherent limitation of sequential and stateful logic families by combining them in a V‐RRAM structure.

Through the synergistic application of both intra‐ and inter‐page logic operations, the proposed architecture enables highly parallelized computations within the V‐RRAM array, as exemplified by large‐scale XOR operations. Building on this configuration, a memristive arithmetic logic unit (mALU) is constructed to perform essential arithmetic operations, including addition, subtraction, increment, and decrement. The mALU, a core component of general‐purpose computing systems, is realized through intrinsic memristive state transitions. This functionality is enabled by the V‐RRAM array's native 3D interconnectivity, which provides both high‐density vertical stacking and page‐level parallelism. Its performance advantages are quantitatively assessed in terms of spatiotemporal cost, demonstrating improved computational throughput and area efficiency. As such, a significant step is taken toward realizing scalable and energy‐efficient in‐memory computing systems based on vertically integrated memristive hardware.

## 2 Results and Discussion

### 2.1 Device Characteristics of Vertical RRAM

**Figure 2:**
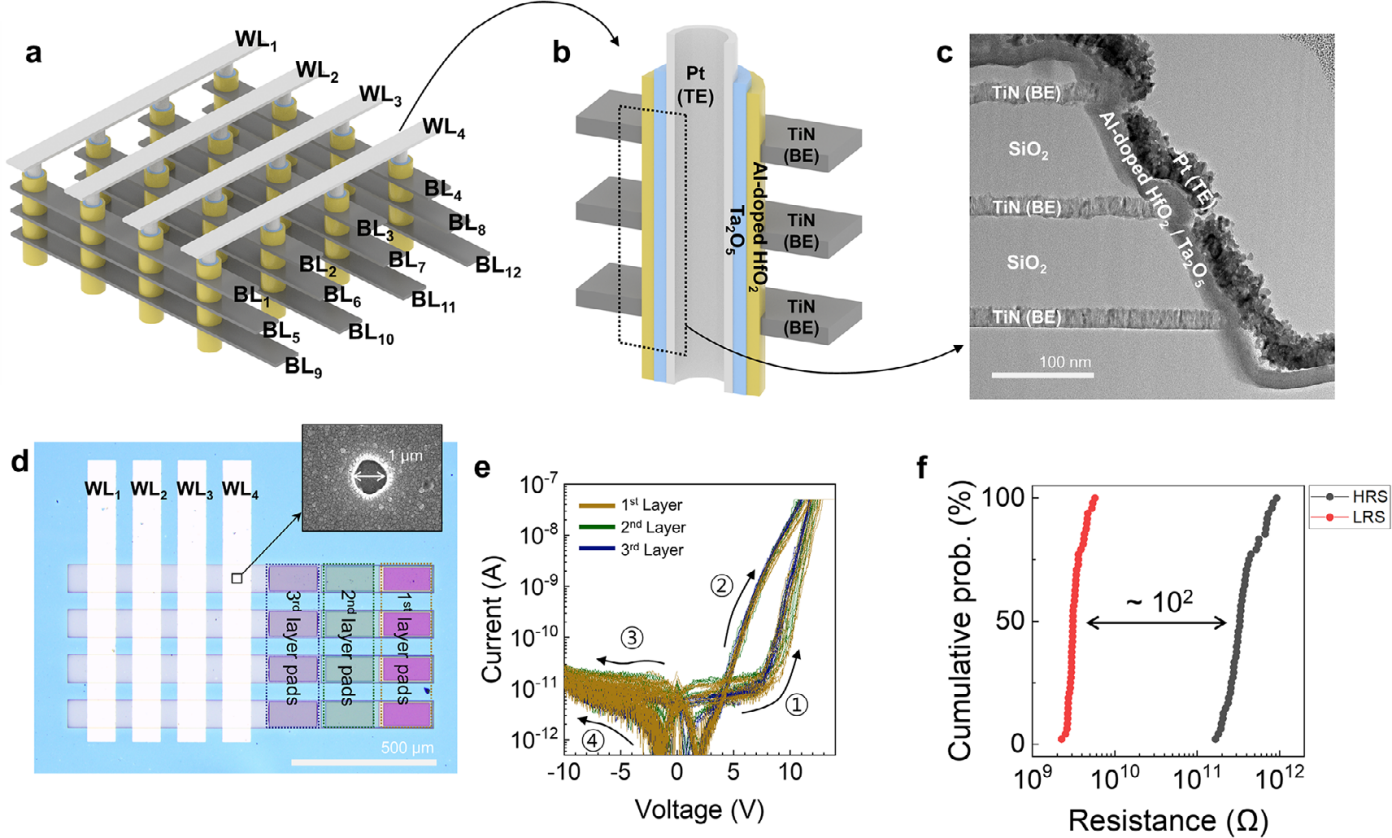
Device structure and electrical characterization of the fabricated three‐layer V‐RRAM array. a) Schematic diagram of the 3D V‐RRAM array layout. b) Cross‐sectional diagram of a single memory hole structure. c) Cross‐sectional TEM image of the vertical stack showing clear interfaces. d) Top‐view optical microscopy image of the V‐RRAM array. The inset shows a top‐view SEM image of a memory hole. e) Uniform self‐rectifying I–V characteristics measured across 48 devices. f) Distribution of HRS and LRS values confirming low D‐to‐D variation and a wide memory window.

**Figure** **2**a shows the schematic diagram of a three‐layer 4 × 4 V‐RRAM array fabricated to enable page‐level logic operations. In this structure, the top and bottom electrodes (TE and BE) correspond to the WLs and BLs, respectively. Figure 2b provides a schematic diagram of a single memory hole structure. The hole‐type V‐RRAM structure was fabricated by etching through a multilayer stack of TiN and SiO_2_, where TiN serves as the BE and SiO_2_ acts as the interlayer dielectric. Following the etching process, the double‐layer resistive switching (RS) layers were sequentially deposited using atomic layer deposition (ALD). The first layer consisted of 10 nm of polycrystalline aluminum‐doped hafnium oxide (Al:HfO_2_), and the second layer was composed of 10 nm of amorphous tantalum pentoxide (Ta_2_O_5_). A platinum (Pt) layer was subsequently deposited to form the TE. As a result, Pt–Ta_2_O_5_–Al:HfO_2_–TiN (PTHT) memristor devices were formed along the sidewalls of the BEs in the first, second, and third memory layers. A detailed fabrication process is described in the Experimental Section and Figure S1 (Supporting Information). The cross‐sectional transmission electron microscopy (TEM) image shown in Figure 2c reveals the internal layering of the three‐layered V‐RRAM structure, clearly distinguishing the TiN, SiO_2_, Al:HfO_2_, Ta_2_O_5_, and Pt. This structure is utilized for the page‐wise logic operations described later. Detailed fabrication procedures are provided in the Experimental Section. Figure 2d shows an optical microscopy image of the V‐RRAM array and a top‐view scanning electron microscopy (SEM) image of the memory hole. The image reveals that the four TE pads are positioned along the upper side of the array, while the twelve BE pads (four per memory layer and three in each memory layer) are located on the right‐hand side. Memory holes with a diameter of 1 µm are located at the intersections of the TE and BE lines. Figure 2e presents representative I–V characteristics measured from 48 devices across the three‐layer array, demonstrating uniform and reliable switching behavior across all layers. The memristors exhibit self‐rectifying properties with low operation current and a high forward‐to‐reverse current ratio. The self‐rectifying characteristics arise from the Schottky barrier formed at the Pt/Ta_2_O_5_ interface, where the high work function of Pt suppresses electron injection when the Pt electrode is negatively biased.^[^ ^31^ ^]^ This self‐rectifying behavior plays a crucial role in suppressing sneak currents in the V‐RRAM structure. A detailed discussion of sneak current suppression, supported by experimental leakage measurements, device modeling, and circuit‐level simulations, is provided in Note S1 (Supporting Information), along with Figures S2–S4 (Supporting Information). The set voltage at which the current reaches the compliance level (50 nA) is ≈12.9 V, while the reset voltage, corresponding to the abrupt transition to the HRS state, is ≈–7 V, as shown in Figure S5 (Supporting Information). Figure 2f summarizes the device‐to‐device (D‐to‐D) variability of the low resistance state (LRS) and high resistance state (HRS), measured at a read voltage of 8 V. The read voltage was chosen as 8 V since the LRS/HRS ratio reaches its maximum at this voltage, as shown in Figure S6 (Supporting Information). The LRS and HRS distributions are well separated, and the memory window spans approximately two orders of magnitude (10^2^), ensuring stable logic operation. For 1 M logic, polarity‐driven switching ensures correctness independent of the absolute resistance contrast. In comparison, for 2 M logic, the applied bias is concentrated on the HRS device, protecting the LRS device and ensuring reliable operation even with an ON/OFF ratio of 10^2^. Details about 1 M, 2 M logic are discussed in the subsequent sections. Additional switching characteristics of PTHT V‐RRAM are provided in the Supporting Information. Figure S7 (Supporting Information) presents representative I–V curves from multiple devices across various positions, while Figure S8 (Supporting Information) shows the retention and reproducibility characteristics.

### 2.2 Intra‐Page Logic Operation

Exclusive OR (XOR) operations are widely utilized in digital logic for applications, such as encryption, arithmetic operations, and error correction. Therefore, many previous studies proposed LIM architectures that implement XOR gates using memristors.^[^ ^20^, ^30^, ^32^, ^33^, ^34^, ^35^ ^]^ The XOR operation between the inputs *p* and *q* (p**⊕**q) can be performed using the following logical expression:

(1) $$p⊕q=\left(p\cdot q\right)\mathrm{RNMP}\left(p+q\right)$$

As described in Equation (1), the XOR operation can be realized by cascading OR, AND, and RNIMP functions. In this study, the OR and AND operations are implemented via intra‐page logic based on 1 M logic units, while the RNIMP operation is realized using inter‐page logic with 2 M logic units. By integrating these operations, page‐wise parallel execution of XOR becomes achievable within the V‐RRAM array.

**Figure 3:**
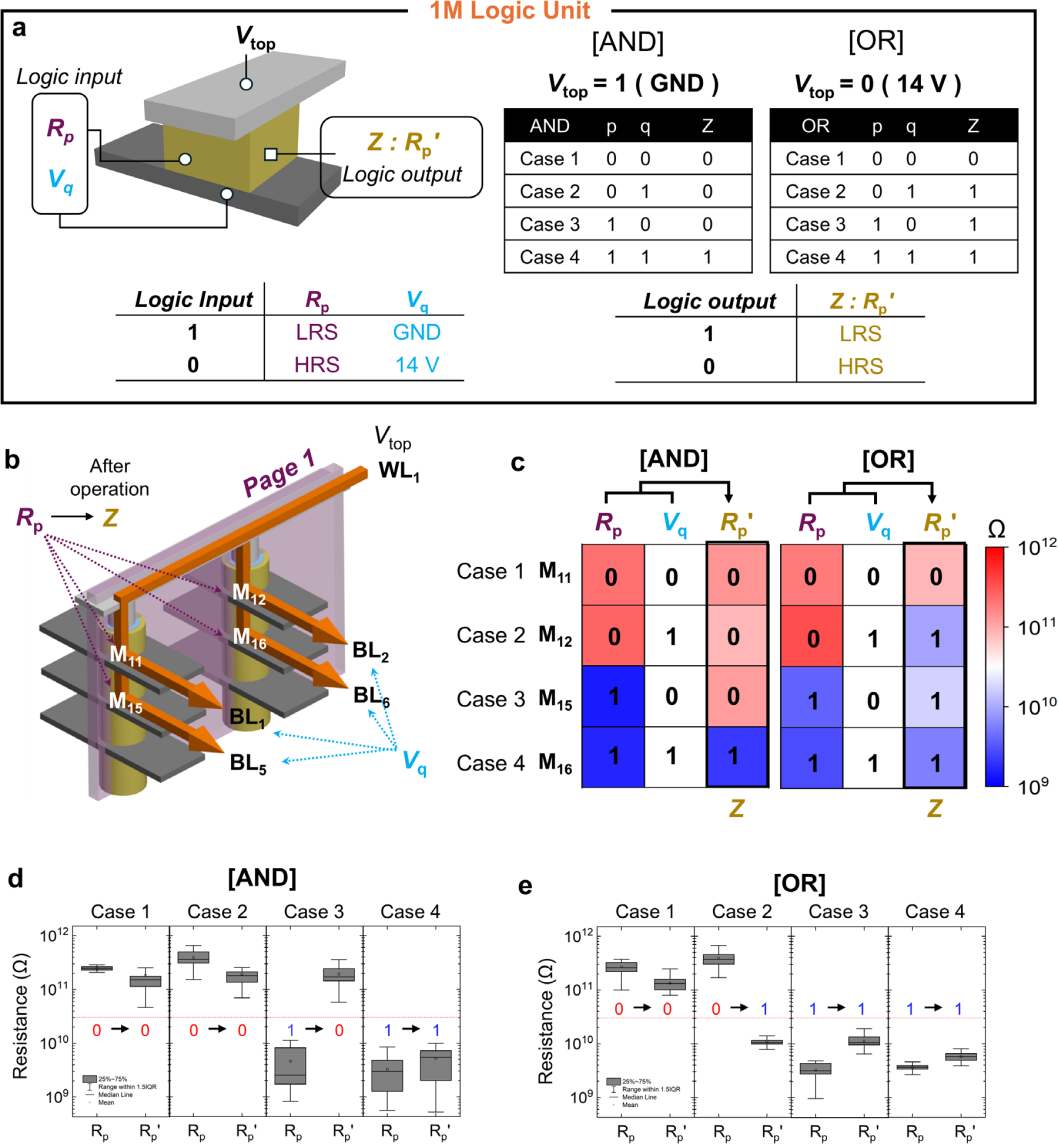
Intra‐page logic operation using 1 M logic. a) Logic configuration for AND and OR gates using single memristors. b) Schematic of intra‐page logic based on V‐RRAM. c) Experimental verification of parallel logic operations across four memristors in a page. d,e) The reproducibilities of d) AND and e) OR gates are shown via boxplot across 50 repetitions.

**Figure** **3**a demonstrates how AND and OR operations can be implemented using 1 M logic and provides a detailed explanation of the input requirements and configuration for each operation. In this study, when data are represented in voltage, logic input data “0” is assigned a value of 14 V (above the switching threshold of the memristor), whereas logic “1” is assigned to the ground potential. When data are represented in resistive states, logic “0” is assigned to the high‐resistance state (HRS, shown in red) and logic “1” to the low‐resistance state (LRS, shown in blue). Depending on the input mode, the logic values are encoded in voltage (V) or resistance (R), with subscripts indicating their logical identity. For instance, an input p represented by voltage is denoted as V_p_, whereas its resistance representation is R_p_. A prime symbol on a data variable (e.g., R_p_′) denotes the result after one logical operation, while a double prime (e.g., R_p_″) indicates that two operations have been sequentially applied. Finally, a bar symbol on a data (e.g., $\bar{p}$) denotes the complement of the data. It should be noted that programming operations (SET/RESET) belong to the information storage phase, whereas Boolean logic functions are executed in a separate computation phase. Because these two phases are decoupled, setting a resistance state does not interfere with subsequent logic operations.

The 1 M logic utilized in this study is based on a structure in which a memristor is pre‐initialized to a resistance state R_p_​ corresponding to input p. A fixed voltage V_top_​ is applied to the TE, and a voltage V_q_ corresponding to input q is applied to the BE. The resulting resistance state of the memristor, denoted as R_p_′, serves as the output of the logic operation. For the 1 M logic gates, the output resistance state is determined by the applied operation voltage of 14 V, which is higher than both the SET and RESET thresholds, together with the biasing scheme of the top electrode (V_top_) and the bottom electrode (V_q_). The initial resistance state of the memristor encodes the input p (HRS = 0, LRS = 1), while the applied BE bias corresponds to the input q, and the voltage drop across the memristor is defined as V_drop_ = V_top_ – V_q_.

For the AND gate, V_top_ is fixed at ground, while the BE voltage is set to 14 V when q = 0 and ground when q = 1. When both inputs are (0, 0), the memristor starts in HRS and experiences V_drop_ = –14 V. Under this bias, no switching occurs, and the memristor remains in HRS, giving output 0. When the inputs are (0, 1), the memristor is initially in HRS, and since V_drop_ = 0 V, no switching occurs, again resulting in output 0. For the case (1, 0), the memristor begins in LRS, and V_drop_ = –14 V exceeds the reset threshold, which switches the device to HRS, yielding output 0. Finally, for (1, 1), the memristor is in LRS and experiences V_drop_ = 0 V, so no switching occurs and the device remains in LRS, corresponding to output 1. Therefore, only the case (1, 1) yields logic 1, reproducing the AND truth table.

For the OR gate, V_top_ is fixed at 14 V, while the BE voltage is set to 14 V when q = 0 and ground when q = 1. When both inputs are (0, 0), the memristor starts in HRS and experiences V_drop_ = 0 V. No switching occurs, and the memristor remains in HRS, yielding output 0. In the case (0, 1), the memristor begins in HRS and V_drop_ = 14 V, which exceeds the set threshold under forward bias, switching the device to LRS and giving output 1. When the inputs are (1, 0), the memristor is initially in LRS and experiences V_drop_ = 0 V, so it remains in LRS and produces output 1. Similarly, for (1, 1), the memristor starts in LRS, and V_drop_ = 14 V is applied, but since the device is already in LRS, it remains unchanged, also producing output 1. Therefore, only the case (0, 0) yields logic 0, consistent with the OR truth table.

Figure 3b presents the schematic diagram of the intra‐page logic architecture based on 1 M logic implemented within a V‐RRAM structure, where memristors connected to a single WL (WL_1_) form a vertically arranged 2D array, referred to as a page (Page 1). When each memristor in the page is pre‐initialized to a resistance state R_p_ corresponding to input p, the application of a fixed voltage (V_top_) to the WL and input‐dependent voltages (V_q_) to each BL activates the intra‐page logic. As a result, a one‐step parallel logic operation is executed across the page, and the computation outcome is stored as an updated resistance state R_p_′ in each memristor. To verify the parallelism capability of the intra‐page logic, Figure 3c shows the experimental results of parallel operations performed on four memristors (M_11_, M_12_, M_15_, and M_16_) within a single page. All resistance values were measured before and after the logic operations using a read voltage of 8 V. The voltages applied to each BL (V_q_) during the logic operations are indicated as white background labels in the accompanying heatmap. Each memristor was initialized to a resistance state corresponding to its respective input p, while a fixed voltage V_top_ was applied to the shared WL_1_, and input‐dependent V_q_ values were simultaneously applied to the BL_1_, BL_2_, BL_5_, and BL_6_. Consequently, all memristors underwent a single‐step logic operation in parallel. Based on a predefined criterion of a resistance value of 3 × 10^10^ Ω, it was confirmed that the correct output resistance state was achieved in all input cases without any errors. Figure 3d,e further demonstrates the reproducibility of intra‐page logic operations by showing box plots of repeated logic executions. In these experiments, the intra‐page AND and OR operations were performed 50 times on the four memristors. The resulting output resistance states were consistently distinguishable according to the predefined criteria, confirming the robustness and repeatability of the intra‐page logic operations. These results experimentally validate that intra‐page logic enables reliable page‐level parallel computation. A more detailed comparison of how the 1 M logic and the intra‐page logic framework address the inherent limitations of conventional stateful and sequential logic—such as variability tolerance, direct cascading, and parallelism—is included in Note S2 (Supporting Information), along with schematic illustrations in Figure S9 (Supporting Information) and a side‐by‐side summary in Table S1 (Supporting Information).

### 2.3 Inter‐Page Logic Operation

In the previously described intra‐page logic, input data were represented by voltage and resistance states, and the results of AND and OR operations were stored as resistance states in a single computational step. Meanwhile, the 2 M logic configuration can extend the parallel operation capability of V‐RRAM inter‐page logic, where both inputs and outputs are encoded in resistance states. A method for implementing 2 M logic using anti‐serially connected self‐rectifying memristors has been previously proposed.^[^ ^30^ ^]^ In this study, the previously reported approach, where logical operations were performed between cells sharing a common TE, was further enhanced by enabling parallel logic operations between cells sharing a common BE. This design enables page‐level parallelism in inter‐page logic.

**Figure 4:**
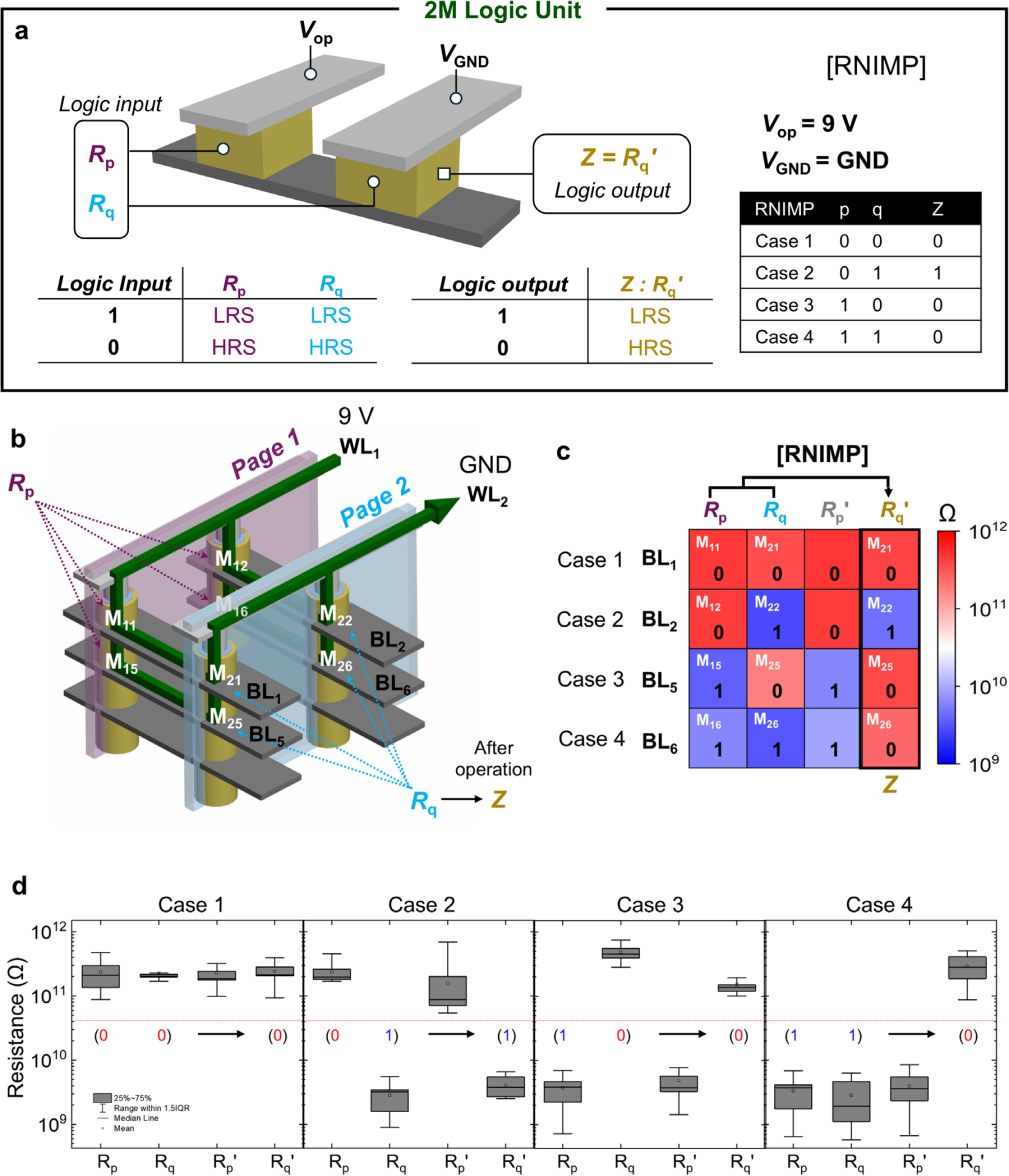
Inter‐page logic operation using 2 M logic. a) Logic configuration of 2 M RNIMP gate using anti‐serial self‐rectifying memristors. b) Schematic of inter‐page logic where two pages interact via shared BLs. c) Experimental verification of inter‐page RNIMP logic across four pairs of memristors. d) Reproducibility of the RNIMP gate shown via box plot across 50 repetitions.

**Figure** **4**a illustrates the configuration of the 2 M logic‐based RNIMP gate, which presents the central idea of the inter‐page logic. For the 2 M logic, two self‐rectifying memristors (storing p and q) are connected in anti‐series with a shared bottom electrode. An operation voltage (V_op_) of 9 V is applied to the top electrode of the memristor storing p, while the top electrode of the memristor storing q is grounded. The logic output is defined as the final resistance state of the q‐memristor. Because of the self‐rectifying property, a memristor under reverse bias behaves effectively as if it were in HRS, which leads to asymmetric voltage division across the two devices. Figures S5 and S10 (Supporting Information) show the reset voltage analysis and device properties. Below explains the case‐by‐case analysis based on these results. When both inputs are (0, 0), meaning both memristors are in HRS, the applied 9 V divides nearly equally and the q‐memristor experiences V_drop_ ≈ –6.5 V, which is below the reset threshold. Thus, R_q_ remains in HRS, and the output is 0. For (0, 1), R_p_ is in HRS and R_q_ in LRS, but under reverse bias, the q‐memristor effectively behaves as HRS, so again the voltage divides nearly equally, giving V_drop_ ≈ –5.7 V. Since this voltage is below the reset threshold, R_q_ stays in LRS, giving output 1. In the case (1, 0), R_p_ is in LRS and R_q_ is in HRS. Because the resistance in the HRS is much larger than that in the LRS, the V_drop_ ≈ –8.9 V, which exceeds the reset threshold. As a result, the q‐memristor either remains or switches to HRS, yielding output 0. Finally, for (1, 1), both memristors are in LRS. The q‐memristor is under reverse bias and V_drop_ ≈ 9 V, which exceeds the reset threshold, so the q‐memristor resets to HRS, resulting in output 0. Collectively, these results confirm that the R_q_ output follows the RNIMP truth table, verifying the correct operation of the 2 M logic primitive.

Figure 4b presents the schematic diagram of the inter‐page logic implemented using 2 M logic, where the WL_1_ and WL_2_ correspond to Pages 1 and 2, respectively. Within each page, memristors are initialized to resistance values R_p_​ and R_q_, which represent input data p and q. During the subsequent logic operation, a fixed operation voltage V_op_ is applied to WL_1_, while WL_2_ is grounded. As a result, for each pair of memristors sharing the same BL, a voltage division occurs across the memristors, triggering the inter‐page logic operation. The operation is completed in a single step, and the result is stored as the updated resistance state R_q_′​ in the memristors located on Page 2. It is readily noted that similar operations can be achieved on other layers, enabling parallel logic operations. Figure 4c shows the experimental validation of inter‐page logic using four memristor pairs (sharing BL_1_ and BL_2_ on the third layer, and BL_5_ and BL_6_ on the second layer) from page 1 and page 2 in a V‐RRAM array. The memristors on Page 1 were initialized to resistance states corresponding to input p, and those on Page 2 were initialized to states corresponding to input q. Subsequently, an operation voltage of 9 V was applied to WL_1_, and WL_2_ was grounded. All four memristor pairs were operated simultaneously, enabling single‐step RNIMP logic computation. The results were read using a single read voltage of 8 V and visualized accordingly. Under the predefined resistance criterion, all input cases yielded the expected logic outputs R_q_′ with no observed errors. Furthermore, it was confirmed that the resistance states of memristors storing input p remained unchanged before and after the operation (R_p_ = R_p_′​). It is also worth noting that the NOT operation is inherently included in the RNIMP primitive. When the input q is fixed to logic “1” (LRS), the RNIMP operation reduces to p̅, thereby directly realizing NOT. This equivalence can be clearly observed in the experimental RNIMP results presented here.

Figure 4d illustrates the reproducibility of the inter‐page RNIMP logic by repeating the operation 50 times across the four memristor pairs. As with the intra‐page operations, all output states could be clearly distinguished according to the predefined resistance criteria, and correct logic results were consistently obtained. These findings experimentally verify the feasibility of parallel inter‐page logic operations based on page‐to‐page interactions. Additional details demonstrating that both 1 M and 2 M logic operations are feasible under pulsed‐voltage bias conditions are provided in Figure S11 and Note S4 (Supporting Information).

### 2.4 Parallel XOR Logic Operation

**Figure 5:**
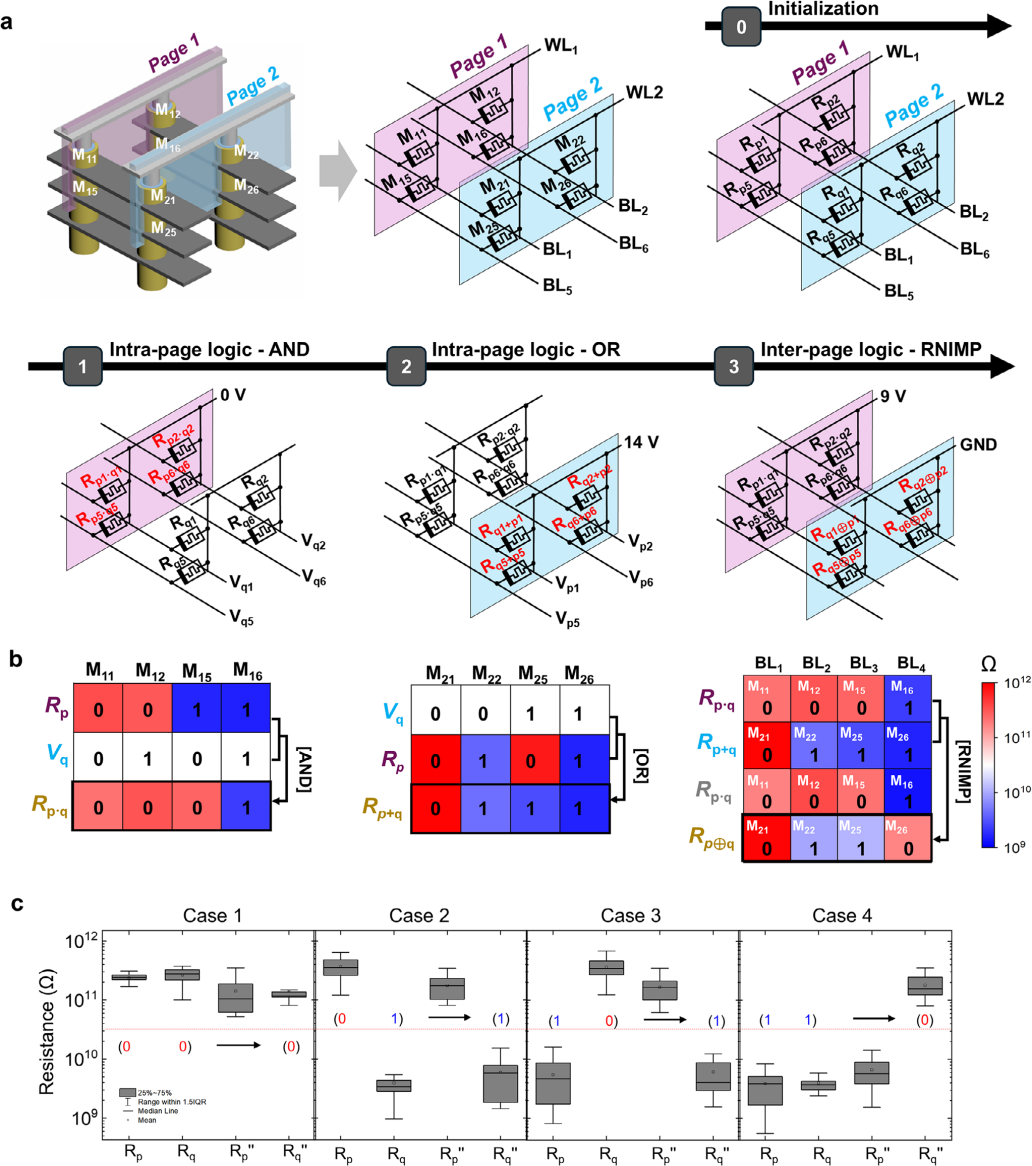
Parallel XOR implementation via cascading intra‐ and inter‐page logic. a) 3‐step execution method for bitwise XOR using V‐RRAM. b) Heatmap results of each logic step: intra‐page AND (left), intra‐page OR (center), and inter‐page RNIMP (right). c) Reproducibility test of 4‐bit XOR operation across four device pairs over 50 repetitions.

The V‐RRAM architecture can further support bitwise‐XOR operations without requiring any additional peripheral circuits or off‐chip computation by applying controlled voltage to the memory array. **Figure** **5**a illustrates the proposed method for obtaining page‐wise XOR results based on the identity in Equation (1).

**Step 0**: This step is an initialization step required for the experiment, but is not needed for logic operation during actual in‐memory computing, where data is already stored. The memristors on pages 1 and 2 are initialized to resistance states corresponding to input p and q, respectively. In this experiment, 4‐bit input vectors were used, with Page 1 initialized to a 4‐bit input p (R_p1_, R_p2_, R_p5_, and R_p6_) and Page 2 to a 4‐bit input q (R_q1_, R_q2_, R_q5_, and R_q6_).
**Step 1**: WL_1_ is grounded (V_top_ = GND), and input q is applied to the BLs. This triggers the intra‐page AND gate operation on Page 1, and the result of p**·**q (bitwise AND) is stored in the resistance states of the memristors on Page 1.
**Step 2**: A high bias voltage (V_top_ = 14 V) is applied to WL_2_ while input p is applied to the BLs, enabling the intra‐page OR gate operation in Page 2. Consequently, the memristors on Page 2 store the result of p**+**q (bitwise OR) in their resistance states.
**Step 3**: A logic operation is performed between Pages 1 and 2 using inter‐page RNIMP gates. Specifically, WL_1_ is biased with V_op_ = 9 V, and WL_2_ is grounded. This inter‐page logic results in the bitwise XOR operation p**⊕**q, stored in the resistance states of the memristors on Page 2.

Through this 3‐step process, bitwise XOR can be executed entirely within the array in a page‐parallel manner. It should be noted that the proposed XOR operation does not require intermediate readouts or additional processing between the cascaded intra‐page and inter‐page logic steps. However, for accurate experimental validation, the resistance states of each memristor were read after each step using an 8 V read voltage, and the results are shown in Figure 5b. The left heatmap displays the result of the intra‐page AND gate operation performed on Page 1. It can be observed that, within a single step, the result of p⋅q is accurately stored in the resistance states (R_p⋅q_​) of the memristors on Page 1. The center heatmap shows the result of the intra‐page OR gate operation performed on Page 2. Similarly, within a single step, the result of p+q is stored in the resistance states (R_p+q_) of the memristors, as shown on Page 2. Finally, the right heatmap shows the result of the inter‐page RNIMP gate operation between Pages 1 and 2. Through a total of three steps, the XOR result (p⊕q) is stored as resistance states (R_p⊕q_)​ in the memristors of Page 2. These results experimentally validate that bitwise XOR operations can be executed at the page level by cascading intra‐page and inter‐page logic operations.

Figure 5c shows the results of repeated XOR gate operations on four pairs of memristors on Pages 1 and 2 to verify the reproducibility of the proposed method. Each operation was repeated 50 times. The results confirmed that all output resistance states corresponded to the correct logic values and were clearly distinguishable based on the predefined criteria. These findings experimentally demonstrate that the XOR gate—requiring page‐level operations and cascading of intra‐ and inter‐page logic—can be reliably executed. If the number of V‐RRAM layers exceeds the bit width of the input, the inputs can be stored and computed in a vertically stacked manner. This enables the XOR operation to be performed using devices with an area of only two cells, regardless of the bit width. Still, they can be executed with the three steps. As a result, the proposed XOR method achieves minimal spatiotemporal cost (STC). **Table** **1** compares the STC of n‐bit XOR operations using this method with those reported in other studies on memristive logic. Owing to the high area efficiency of PTHT V‐RRAM and the high parallelism of page‐level logic operations, the proposed approach significantly reduces the STC of bitwise XOR operations. This suggests that the proposed method offers substantial advantages for XOR‐based LIM tasks, such as encryption and similarity comparison. In addition to the spatiotemporal benefits, the page‐wise scheme inherently offers higher throughput than conventional 2D memristor arrays due to its reduced step count. While the immediate energy reduction is minimal with the present device characteristics, the advantage is expected to grow as lower power memristors are developed, where peripheral overhead becomes the dominant factor (see Note S5 and Figures S15 and S16, Supporting Information, for detailed benchmarking).

**Table 1:** Comparison of spatiotemporal cost for N‐bit XOR operations using the proposed scheme and previously reported memristor‐based logic methods. The proposed method achieves significantly lower cost due to its constant 3‐step execution and 2‐cell footprint, enabled by 3D stacking and page‐wise parallelism.

Reference	Y. R. Kim et al.^[^ ^20^ ^]^	L. Xu et al.^[^ ^36^ ^]^	L. Yang et al.^[^ ^37^ ^]^	K. S. Woo et al.^[^ ^38^ ^]^	This work
Memristor type	Ta/HfO_2_/RuO_2_	TiN/Ta_2_O_5_/SiO_2_/Ta_2_O_5_/TiN	Ti/HfO_2_/TiN	Cu_x_Te_1‐x_/HfO_2_/Pt	Pt/Ta_2_O_5_/Al:HfO_2_/TiN
Technology	Passive array	Passive array	1T1R	Passive array	Passive array
Vertically integrated structure	X	X	X	X	O
Footprint area for N‐bit XOR operation (unit = cells) (A)	3N	N^2^	N	N^2^	2
Number of steps for N‐bit XOR operation (B)	1	2	N	3	3
STC for 8‐bit XOR operation (N = 8, A × B)	24	128	64	192	6

### 2.5 Memristive Arithmetic Logic Unit

**Figure 6:**
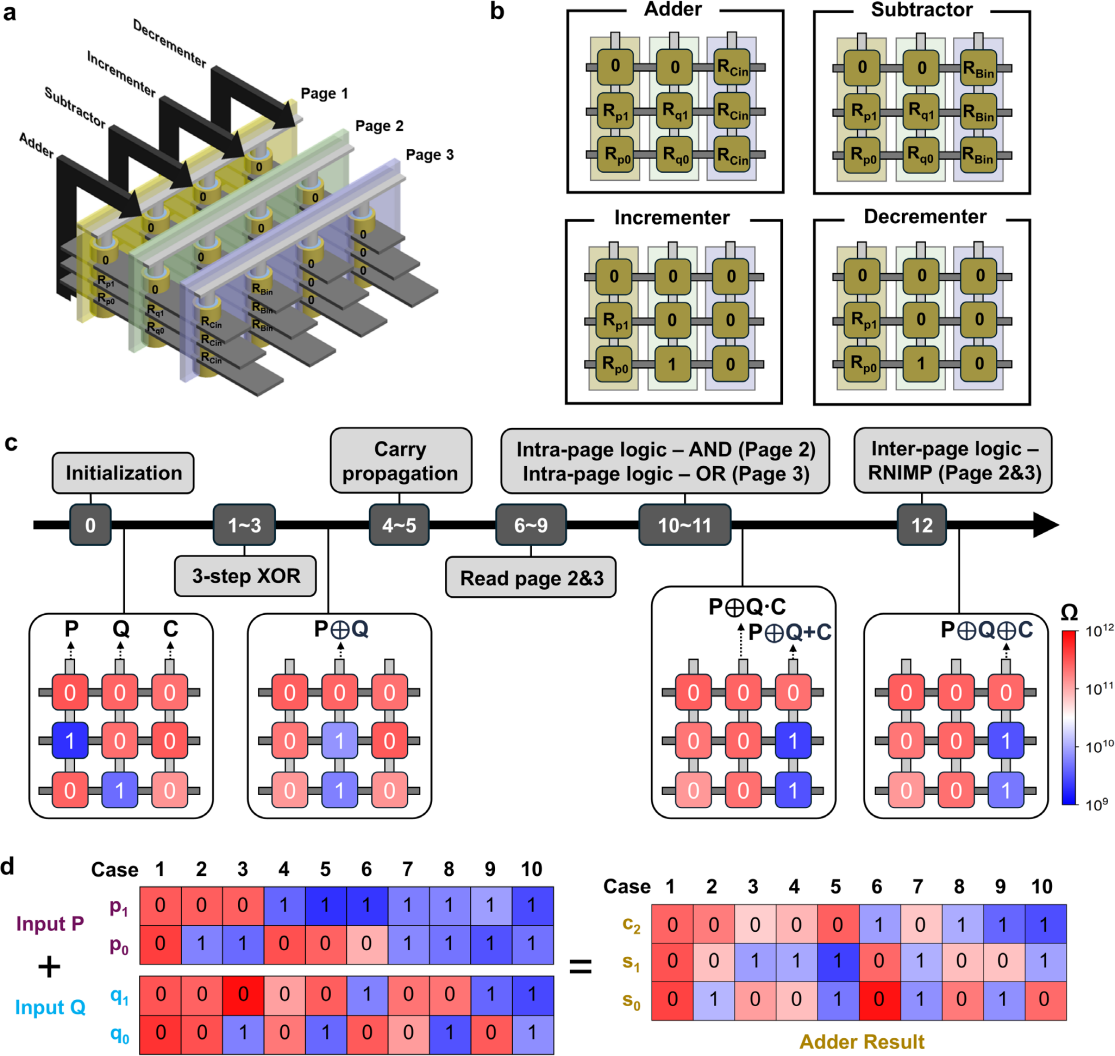
Implementation of a 2‐bit mALU. a) Schematic of a 2‐bit mALU composed of four arithmetic units on a 3‐layer V‐RRAM stack. b) Initialization method for adder, subtractor, incrementer, and decrementer. c) Step‐by‐step experimental verification of 10 + 01 = 11 using the proposed 2‐bit full adder method. d) Experimental results of the 2‐bit full adder scheme confirming the correct output of all input combinations.

The ALU is a core component of modern processors, enabling essential operations such as AND, OR, addition, subtraction, and others, which are executed separately from memory in conventional systems, thereby incurring significant energy and latency overheads. This work proposes a method for constructing an mALU using the previously defined intra‐ and inter‐page logic primitives. Each arithmetic function is realized by reconfiguring voltage inputs within the given array structure, enabling compact and parallel execution. The mALU enables the direct implementation of combined arithmetic operations within the memory array, offering significant advantages in terms of area and energy efficiency when processing large volumes of data. The experimentally demonstrated AND, OR, XOR, and RNIMP gates, along with the NOT functionality inherently provided by RNIMP, complete the universal Boolean function set supported by our 3D vertical logic‐in‐memory architecture. Utilizing this property, arithmetic operations such as addition, subtraction, increment, and decrement can also be constructed entirely by XOR and majority logic gates. These arithmetic functions can be expressed using the following logical equations:

(2) $$p_{n}⊕q_{n}⊕C_{n}=S_{n},\;p_{n}⊕q_{n}⊕B_{n}=D_{n}$$

(3) $$\mathrm{MAJ}\left(p_{n},\;q_{n},\;C_{n}\right)=C_{n+1},\;\;\mathrm{MAJ}\left({\bar{p}}_{n},\;q_{n},\;B_{n}\right)=B_{n+1}$$

where *n* denotes the bit index, *C* is the carry, *S* is the sum, *B* is the borrow, *D* is the difference, and *MAJ* refers to the majority gate logic operation. In the V‐RRAM hardware used in this work, the proposed 3‐step XOR operation can be executed twice to realize the sum or difference operation. Furthermore, the majority gate can also be implemented using memristive logic.^[^ ^34^ ^]^ **Figure** **6**a shows a schematic diagram of a 2‐bit mALU composed of four arithmetic units implemented on a 3‐layer V‐RRAM stack. Each arithmetic unit operates in the direction indicated by the black arrows in the figure. Because the 3‐step XOR operation is performed at the page level, all XOR computations within the arithmetic units can be executed in parallel. Likewise, carry signals can be propagated in parallel across all four units, as discussed later. Figure 6b illustrates the initialization method for activating each arithmetic unit. For the adder, two 2‐bit inputs, p and q, are stored along the vertical direction as resistance states in memristors located on pages 1 and 2, respectively. Any remaining memristors in the 3‐layer structure are initialized to 0. All memristors on Page 3 are initialized to the resistance state corresponding to the carry‐in (*C_in_*). For the subtractor, the difference is computed similarly to the sum, and the *B* can be propagated by applying a negated voltage corresponding to input p. Therefore, the initialization procedure is identical to that of the adder. An incrementer can be implemented by configuring the adder with input q set to 01 and carry‐in to 0. In contrast, a decrementer can be implemented by configuring the subtractor with q set to 01 and borrow‐in to 0. Figure 6c presents the implementation of the 2‐bit full adder using the page‐wise logic, along with experimental step‐by‐step data demonstrating the computation of 10 + 01 = 11. Here, each step is not merely defined by a logical operation but rather by a complete sequence involving the application of specific voltages, the resulting resistance changes induced by switching, and the subsequent readout of those resistance states. Each of these processes is counted and represented individually in the step index to reflect the physical progression of the computation accurately.

**Step 0**: Initialization is performed according to the Adder method described in Figure 6b.
**Steps 1–3**: A 3‐step XOR operation is executed between pages 1 and 2 using the method described in the previous section. As a result, the XOR output of p and q is stored as resistance states on page 2. At this stage, the third‐layer memristors store the result of 0**⊕**0, so their resistance states remain unchanged.
**Steps 4–5**: Carry is propagated. Since the proposed 2‐bit full adder is a ripple‐carry adder, the carry is propagated sequentially at each bit using a sequential logic approach based on Equation (3). Note S6 and Figure S12 (Supporting Information) provide details and circuit diagrams illustrating the carry propagation method using the majority gate principle. Specifically, memristors pre‐initialized to the resistance state representing the carry‐in receive the complement of q as a voltage at the TE and p as a voltage at the BE. This sequential logic operation realizes a majority gate, and the resulting carry‐out value is stored in the resistance state of the memristor.
**Steps 6–9**: The XOR results p**⊕**q stored on page 2 and the carry values C_0_ and C_1_ stored on page 3 are sequentially read out. This process requires 2*n* steps for *n*‐bit inputs.
**Step 10**: WL_2_ is grounded, and voltages corresponding to C_1_ and C_0_ are applied to BL_5_ and BL_9_, respectively, to activate the intra‐page AND gate on page 2.
**Step 11**: 14 V is applied to WL_3_, while voltages corresponding to p_1_ **⊕**q_1_ and p_0_ **⊕**q_0_ are applied to BL_5_ and BL_9_, respectively, to activate the intra‐page OR gate on page 3.
**Step 12**: Finally, the operation voltage (V_op_) is applied to WL_2_, and WL_3_ is grounded to activate the inter‐page RNIMP gate between pages 2 and 3. This stores the final sum result of p and q on page 3.

During this final step, since the third‐layer memristor on page 3 performs the sum operation 0**⊕**0**⊕**C_2_, the overflow C_2_ is preserved in its resistance state (R_c2_). Figure 6d presents the experimental validation results for the proposed 2‐bit full adder, showing the performance for various input combinations. The final readout of input and output values confirms that, in all cases, the sum of p and q is accurately stored in the memristors as resistance states.

In this method, since the information is stored and computed in a vertically stacked manner, the projected area occupied by the full adder corresponds to only 3 cells, resulting in excellent area efficiency. The mALU, implemented using page‐level XOR operations and parallel carry propagation, occupies a total projected area of only 12 cells (parallel carry propagation is described in detail in Note S6, Tables S2 and S3, and Figure S13, Supporting Information). The results of each mALU operation—including the sum and difference of inputs p and q, as well as the increment and decrement of p—are produced on page 3 and are vertically aligned within the memristor array.

**Table** **2** presents a comparison between the proposed n‐bit full adder method and previously reported works. In the proposed method, the steps required for carry propagation and data readout scale linearly with the number of bits N, while all other operations are performed in parallel, regardless of bit width. As a result, the total number of steps is proportional to 3N. Furthermore, since the input data are stored and processed along the vertical direction, the required projected area is fixed at only 3 cells. Based on the high area efficiency of the 3D V‐RRAM characteristics, this work achieves a significantly lower STC compared to previous approaches.

**Table 2:** Comparison of N‐bit full adder implementations in terms of structure, logic type, number of steps, footprint area, and overall STC. The proposed method outperforms others by utilizing a vertically stacked structure and page‐wise operation.

Reference	Y. S. Kim et al.^[^ ^39^ ^]^	Y. Song et al.^[^ ^2^ ^]^	T. Park et al.^[^ ^34^ ^]^	M. G. Choi et al.^[^ ^40^ ^]^	This work
Adder type	Carry look‐ahead adder	Carry look‐ahead adder	Ripple carry adder	Parallel prefix adder (Kogge‐Stone adder)	Ripple carry adder
Logic state variable	Resistance	Resistance & voltage	Resistance & voltage	Resistance & voltage	Resistance & voltage
Number of steps for N‐bit full adder (A)	5N + 3	3N	2N + 1	5log_2_N + 1	3N + 6
Footprint area for N‐bit full adder (unit: cells) (B)	13N	9N‐9	4N + 1	2Nlog_2_N + 4N	3
STC for 8‐bit full adder (A × B, N = 8)	4472	1512	561	1280	90
3D structure	X	X	X	X	O

## 3 Conclusion

This study proposed and experimentally demonstrated a novel LIM architecture capable of page‐wise logic operations, implemented on a vertically stacked 3D V‐RRAM array composed of Pt–Ta_2_O_5_–Al:HfO_2_–TiN‐based self‐rectifying memristors. Two logic primitives, referred to as 1 M and 2 M logic, were developed to enable both intra‐page and inter‐page logic operations through controlled resistive switching, allowing fundamental Boolean logic functions to be executed directly within the memory array. Building upon this capability, an mALU capable of performing addition, subtraction, increment, and decrement operations was designed. A 2‐bit full adder was feasibly implemented using only three vertically stacked cells and completed its operation in 12 sequential steps, demonstrating high reproducibility and operational stability. Compared to conventional LIM approaches, the proposed method exhibited significantly lower spatiotemporal cost owing to its compact 3D cell structure and massively parallel, page‐wise computation capability. By integrating the strengths of stateful and sequential logic while eliminating their inherent trade‐offs, the 1 M logic framework provides a robust and scalable foundation for practical in‐memory computation. Furthermore, by unifying memory and logic within the given physical location, this non‐von Neumann system architecture offered the potential to diminish data transfer overhead, which is inherently present in conventional CMOS‐based computing platforms. Importantly, this method represents a fundamentally different computational paradigm from conventional CMOS‐based spatial logic. The CMOS CPUs and GPUs are restricted to executing fixed operations defined at design time. In contrast, the proposed memristor‐based logic framework offered reconfigurability, enabling logic functions to be dynamically altered through programmable resistance states. This inherent flexibility enabled the implementation of adaptive logic within the same physical hardware, providing reconfigurable computational resources that could be tailored to specific workload requirements. By utilizing the vertical integration of memristor arrays, the system achieved high integration density and supported large‐scale parallel operations that were challenging with conventional 2D CMOS circuits. This functionality enabled genuine in‐memory computation within a 3D structure, making the architecture particularly well‐suited for applications that require localized, high‐throughput, and repetitive operations across multiple parallel cells, such as edge computing, in‐sensor processing, similarity‐based search, and approximate reasoning.

While memristor‐based logic is not intended to fully replace CMOS logic, it holds significant promise as a complementary processing resource capable of offloading specific computational workloads. In particular, it can efficiently handle certain operations that are less suited to conventional processors by exploiting in‐memory parallelism, thereby reducing system‐level energy consumption and improving response time. This work proposed a viable pathway toward hybrid computing architectures, in which memristive logic was integrated with CMOS systems to accelerate future computing platforms cooperatively. Importantly, the page‐wise parallel logic methodology presented is not limited to the specific V‐RRAM stack demonstrated in this work but constitutes a generalizable framework that can be extended to more advanced memristive technologies as they emerge. In this sense, the proposed approach offers a versatile and forward‐looking route toward scalable 3D logic‐in‐memory systems across diverse resistive switching platforms.

## 4 Experimental Section

### Device Fabrication

A three‐layered vertical 4 × 4 V‐RRAM array was constructed through the following sequence. Initially, a 30 nm‐thick TiN film was deposited onto a SiO_2_/Si substrate using radio frequency (RF) sputtering in a nitrogen environment (Sorona, SRN 120) with a Ti metal target. Subsequently, a 100 nm‐thick SiO_2_ layer was deposited via plasma‐enhanced chemical vapor deposition (PECVD, Oxford Instruments, PlasmaPro System100) to serve as the dielectric layer between cells. This deposition process was repeated three times to build the full three‐layered stack. Patterning was carried out using a maskless lithography tool (Nano System Solutions Inc., DL‐1000 HP). Afterward, the multilayer structure was etched into 100 µm‐wide line patterns using a dry etching system (GIGALANE, NeoS‐MAXIS 200L). Post‐etch residues were removed using a photoresist asher (plasma finish, V15‐G). To access the bottom electrodes at each layer, TiN in the contact pad regions was selectively removed via dry etching (Oxford Instruments, PlasmaPro System100 Cobra), and the underlying SiO_2_ was etched using buffered oxide etchant (BOE) to form stepwise contact openings. A 60 nm‐thick SiO_2_ passivation layer was then deposited by PECVD to prevent unintended device formation on the structure's sidewalls. Circular memory holes, 1 µm in diameter, were defined via photolithography and subsequently etched through the multilayer stack using the same dry etching process. Residual photoresist was removed by O_2_ plasma ashing, and a buffered oxide etchant (BOE) pre‐clean was then applied to strip the TiON interlayer formed on TiN during the ashing step. Further details of the BOE pre‐clean are provided in Figure S14 (Supporting Information). RS layers were deposited inside the holes through ALD. A 10 nm‐thick aluminum‐doped HfO_2_ (Al:HfO_2_) layer was first deposited at 280 °C using thermal ALD (CN1, custom cluster system), with Hf(NCH_3_C_2_H_5_)_4_ and O_3_ as precursors. Aluminum doping was achieved by co‐depositing Al(CH_3_)_3_ at a cycle ratio of 1:9 (Al_2_O_3_:HfO_2_). This was followed by a 10 nm‐thick Ta_2_O_5_ layer deposited at 200 °C using plasma‐enhanced ALD (CN1, Atomic Premium plus 200) with (CH_3_)_3_CNTa(N(C_2_H_5_)_2_) and plasma‐activated H_2_O. Next, a 50 nm‐thick platinum (Pt) top electrode was deposited by electron‐beam evaporation (SRN‐200, SORONA) and patterned using a lift‐off process. To finalize the device, any RS material remaining above the contact pads was removed by dry etching, and the exposed SiO_2_ passivation was etched away using BOE to complete the pad formation.

### Electrical Measurements

The I–V characteristics of the V‐RRAM devices were evaluated using a semiconductor parameter analyzer (SPA, HP 4155A). During I–V measurements, a bias voltage was applied to the top electrode while the bottom electrode was grounded. For measuring intra‐plane and inter‐plane logic operations at the array level, a custom‐designed multiprobe setup was used to contact equally spaced pads, allowing simultaneous access to multiple devices. A switch matrix (Keithley 708A) was used to address target cells for logic operations selectively, and the SPA (HP 4155B) was employed to apply the required voltage pulses. For state readout, individual cells were sequentially selected through the switch matrix, and a read voltage of 8 V was applied to extract the mapped resistance states. Due to the self‐rectifying nature of the array, only the resistance of the selected cell could be reliably read without interference from adjacent unselected cells.

## Supporting information



Supporting Information

## Data Availability

The data that support the findings of this study are available from the corresponding author upon reasonable request.

## References

[1] J. Von Neumann , IEEE Annals History Comput. 1993, 15, 27.

[2] Y. Song , X. Wang , Q. Wu , F. Yang , C. Wang , M. Wang , X. Miao , Adv. Sci. 2022, 9, 2200036. 10.1002/advs.202200036 PMC9130921 35343097

[3] H. A. Du Nguyen , L. Xie , M. Taouil , R. Nane , S. Hamdioui , K. Bertels , IEEE Trans. Very Large Scale Integr. VLSI Syst. 2017, 25, 2206.

[4] N. Xu , T. Park , K. J. Yoon , C. S. Hwang , phys. status solidi (RRL)–Rapid Res. Lett. 2021, 15, 2100208.

[5] F. Wei , X. Cui , X. Cui , IEEE J. Electron Devices Soc. 2019, 8, 57.

[6] Y. Zhou , Y. Li , N. Duan , Z. Wang , K. Lu , M. Jin , L. Cheng , S. Hu , T. Chang , H. Sun , Adv. Electron. Mater. 2018, 4, 1800229.

[7] E. Linn , R. Rosezin , S. Tappertzhofen , U. Böttger , R. Waser , Nanotechnology 2012, 23, 305205. 22782173 10.1088/0957-4484/23/30/305205

[8] A. Siemon , R. Drabinski , M. J. Schultis , X. Hu , E. Linn , A. Heittmann , R. Waser , D. Querlioz , S. Menzel , J. S. Friedman , Sci. Rep. 2019, 9, 14618. 31602003 10.1038/s41598-019-51039-6 PMC6787102

[9] J. Borghetti , G. S. Snider , P. J. Kuekes , J. J. Yang , D. R. Stewart , R. S. Williams , Nature 2010, 464, 873. 20376145 10.1038/nature08940

[10] N. Xu , K. J. Yoon , K. M. Kim , L. Fang , C. S. Hwang , Adv. Electron. Mater. 2018, 4, 1800189.

[11] S. Yu , B. Gao , Z. Fang , H. Yu , J. Kang , H. P. Wong , Adv. Mater. 2013, 25, 1774. 23355110 10.1002/adma.201203680

[12] S. Pi , C. Li , H. Jiang , W. Xia , H. Xin , J. J. Yang , Q. Xia , Nat. Nanotechnol. 2019, 14, 35. 30420759 10.1038/s41565-018-0302-0

[13] B. J. Choi , A. C. Torrezan , J. P. Strachan , P. G. Kotula , A. J. Lohn , M. J. Marinella , Z. Li , R. S. Williams , J. J. Yang , Adv. Funct. Mater. 2016, 26, 5290.

[14] Y. Zhong , J. Tang , X. Li , B. Gao , H. Qian , H. Wu , Nat. Commun. 2021, 12, 408. 33462233 10.1038/s41467-020-20692-1 PMC7814066

[15] C. Du , F. Cai , M. A. Zidan , W. Ma , S. H. Lee , W. D. Lu , Nat. Commun. 2017, 8, 2204. 29259188 10.1038/s41467-017-02337-y PMC5736649

[16] J. Moon , W. Ma , J. H. Shin , F. Cai , C. Du , S. H. Lee , W. D. Lu , Nat. Electron. 2019, 2, 480.

[17] S. Wen , H. Wei , Z. Yan , Z. Guo , Y. Yang , T. Huang , Y. Chen , IEEE Trans. Netw. Sci. Eng. 2019, 7, 1431.

[18] Z. Liu , J. Tang , B. Gao , P. Yao , X. Li , D. Liu , Y. Zhou , H. Qian , B. Hong , H. Wu , Nat. Commun. 2020, 11, 4234. 32843643 10.1038/s41467-020-18105-4 PMC7447752

[19] P. Yao , H. Wu , B. Gao , J. Tang , Q. Zhang , W. Zhang , J. J. Yang , H. Qian , Nature 2020, 577, 641. 31996818 10.1038/s41586-020-1942-4

[20] Y. R. Kim , D. H. Shin , N. Ghenzi , S. Cheong , J. M. Cho , S. K. Shim , J. K. Lee , B. S. Kim , S. Yim , T. Park , Adv. Funct. Mater. 2025, 35, 2414332.

[21] S. Kvatinsky , D. Belousov , S. Liman , G. Satat , N. Wald , E. G. Friedman , A. Kolodny , U. C. Weiser , IEEE Trans. Circuits Syst. II: Exp. Briefs 2014, 61, 895.

[22] L. Gao , F. Alibart , D. B. Strukov , IEEE Trans. Nanotechnol. 2013, 12, 115.

[23] S. Kvatinsky , G. Satat , N. Wald , E. G. Friedman , A. Kolodny , U. C. Weiser , IEEE Trans. VLSI Syst. 2013, 22, 2054.

[24] H. Li , K.‐S. Li , C.‐H. Lin , J.‐L. Hsu , W.‐C. Chiu , M.‐C. Chen , T.‐T. Wu , J. Sohn , S. B. Eryilmaz , J.‐M. Shieh , In IEEE Symp. on VLSI Technology , IEEE, New York 2016, pp. 1.

[25] Q. Luo , X. Xu , H. Liu , H. Lv , T. Gong , S. Long , Q. Liu , H. Sun , W. Banerjee , L. Li , In 2015 IEEE Int. Electron Devices Meeting (IEDM) , IEEE, New York 2015, pp. 10.

[26] X. Xu , Q. Luo , T. Gong , H. Lv , S. Long , Q. Liu , S. S. Chung , J. Li , M. Liu , In 2016 IEEE Symp. on VLSI Technology , IEEE, New York 2016, pp. 1.

[27] S. S. Kim , S. K. Yong , J. Kim , J. M. Choi , T. W. Park , H. Y. Kim , H. J. Kim , C. S. Hwang , Adv. Electron. Mater. 2023, 9, 2200998.

[28] K. Jeon , J. J. Ryu , S. Im , H. K. Seo , T. Eom , H. Ju , M. K. Yang , D. S. Jeong , G. H. Kim , Nat. Commun. 2024, 15, 129. 38167379 10.1038/s41467-023-44620-1 PMC10761713

[29] T. Park , S. S. Kim , B. J. Lee , T. W. Park , H. J. Kim , C. S. Hwang , Nanoscale 2023, 15, 6387. 36919469 10.1039/d3nr00271c

[30] J. M. Cho , S. S. Kim , T. W. Park , D. H. Shin , Y. R. Kim , H. J. Park , D. Y. Kim , S. H. Lee , T. Park , C. S. Hwang , Nanoscale Horiz. 2024, 10, 113. 39535020 10.1039/d4nh00420e

[31] J. H. Yoon , S. J. Song , I. Yoo , J. Y. Seok , K. J. Yoon , D. E. Kwon , T. H. Park , C. S. Hwang , Adv. Funct. Mater. 2014, 24, 5086.

[32] Y. Song , Q. Wu , X. Wang , C. Wang , X. Miao , IEEE Electron Device Lett. 2021, 42, 1398.

[33] X. Wang , H. Deng , W. Feng , Y. Yang , K. Chen , In 2016 35th Chinese Control Conf. (CCC) , IEEE, New York 2016, 5847.

[34] T. Park , Y. R. Kim , J. Kim , J. Lee , C. S. Hwang , Adv. Intell. Syst. 2022, 4, 2100267.

[35] T. Park , Y. R. Kim , D. H. Shin , B. J. Lee , C. S. Hwang , Adv. Intell. Syst. 2023, 5, 2200341.

[36] L. Xu , R. Yuan , Z. Zhu , K. Liu , Z. Jing , Y. Cai , Y. Wang , Y. Yang , R. Huang , Adv. Mater. Technol. 2019, 4, 1900212.

[37] L. Yang , L. Cheng , Y. Li , H. Li , J. Li , T. Chang , X. Miao , Adv. Electron. Mater. 2021, 7, 2001182.

[38] K. S. Woo , J. Han , S. Yi , L. Thomas , H. Park , S. Kumar , C. S. Hwang , Nat. Commun. 2024, 15, 3245. 38622148 10.1038/s41467-024-47488-x PMC11018740

[39] Y. S. Kim , M. W. Son , H. Song , J. Park , J. An , J. B. Jeon , G. Y. Kim , S. Son , K. M. Kim , Adv. Intell. Syst. 2020, 2, 1900156.

[40] M. G. Choi , J. H. In , H. Song , G. Kim , H. Rhee , W. Park , K. M. Kim , Mater. Horiz. 2024, 12, 131. 10.1039/d4mh01196a 39436699

