# Supplementary material for: Vertical Self‐Rectifying Memristive Arrays for Page‐Wise Parallel Logic and Arithmetic Processing
Source: Adv Mater. 2025 Nov 24;38(8):e14099. doi: 10.1002/adma.202514099 (PMC12878806; doi:10.1002/adma.202514099)
Supplement: Supplementary file 1 — Supporting Information [file ADMA-38-e14099-s001.docx]

SUPPLEMENTARY INFORMATION

**Vertical Self-Rectifying Memristive Arrays for Page-Wise Parallel Logic and Arithmetic Processing**

*Kunhee Son^1,†^, Jea Min Cho^1,†^, Dong Hoon Shin^1,†^, Yeong Rok Kim^1^, Néstor Ghenzi^1,2^, Sunwoo Cheong^1^, Byeong Su Kim^1^, Jung Kyu Lee^1^, Sungho Kim^1^, Wonho Choi^1^, Soo Hyung Lee^1^, Janguk Han^1^, and Cheol Seong Hwang^1,^**

^1^Department of Materials Science and Engineering and Inter-university Semiconductor Research Center, College of Engineering, Seoul National University, Seoul, 08826, Republic of Korea

^2^Universidad de Avellaneda UNDAV and Consejo Nacional de Investigaciones Científicas y Técnicas (CONICET), Mario Bravo 1460, Avellaneda, Buenos Aires 1872, Argentina

^†^ These authors contributed equally to this work.

* Corresponding author (e-mail : cheolsh@snu.ac.kr)

**Table of contents**

- **Supplementary Figures S1-S16**
- **Supplementary Table S1-S4**
- **Supplementary Note S1-S6**
- **Supplementary References**

Supplementary Figures


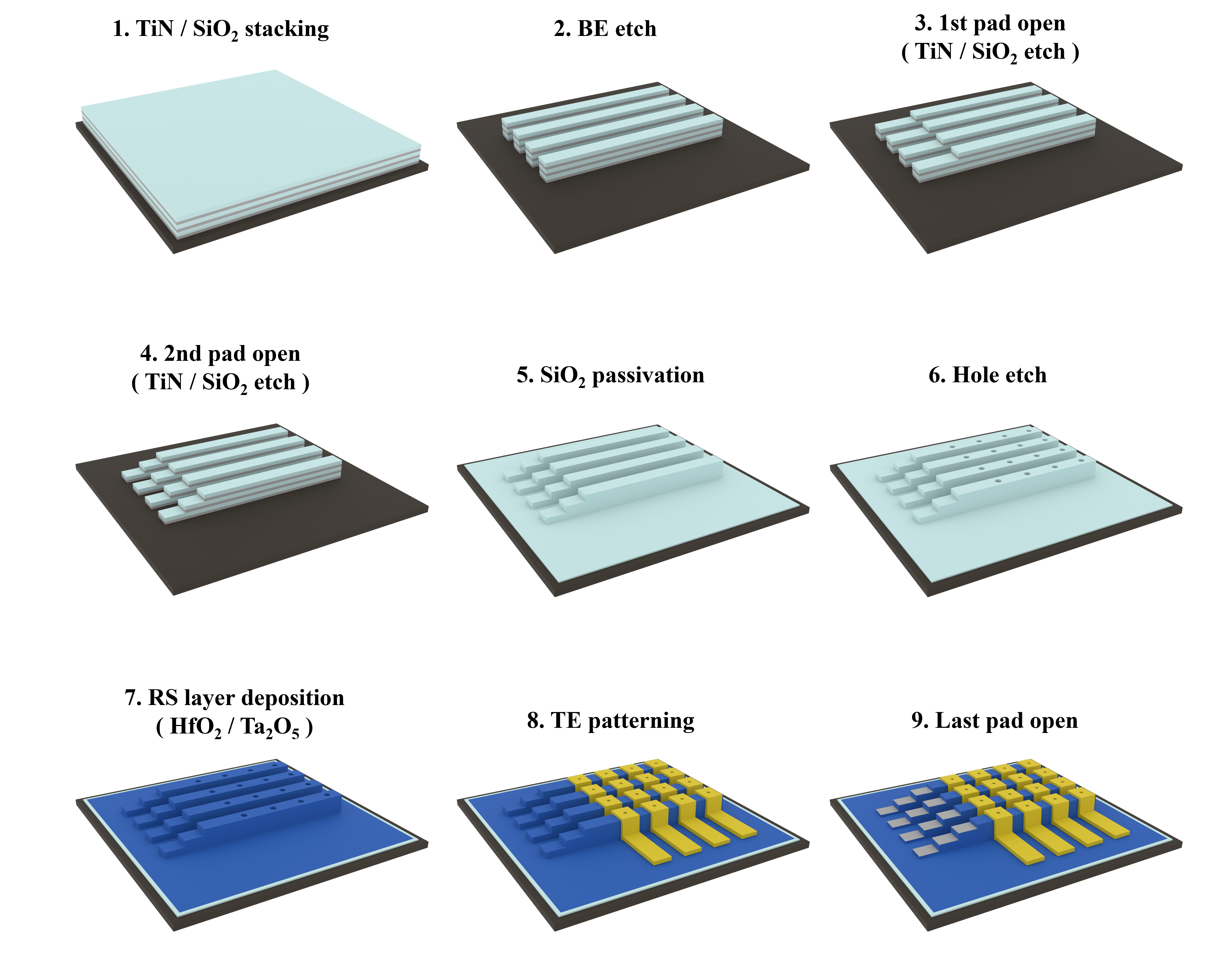


Figure S1 | The schematic diagram showing the fabrication process of the three-layer V-RRAM array (or V-RRAM).


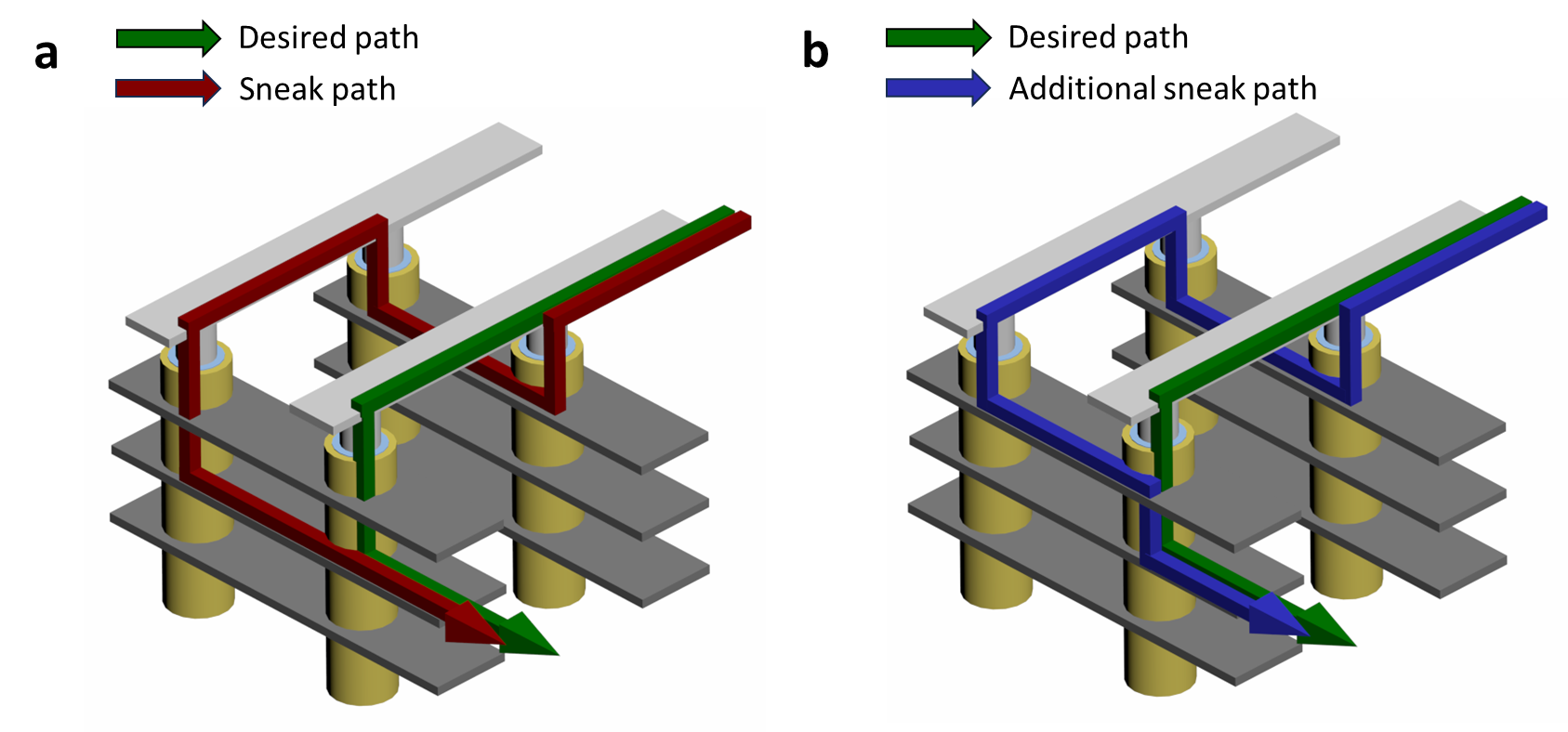

**Figure S2 | Schematic illustration of sneak path formation in the V-RRAM array.**

(a) Desired read current path (green arrows) and undesired sneak paths (red arrows) formed across pages and layers in the V-RRAM structure. (b) Additional vertical sneak paths (blue arrows) that may form between adjacent bit lines due to inter-layer leakage.


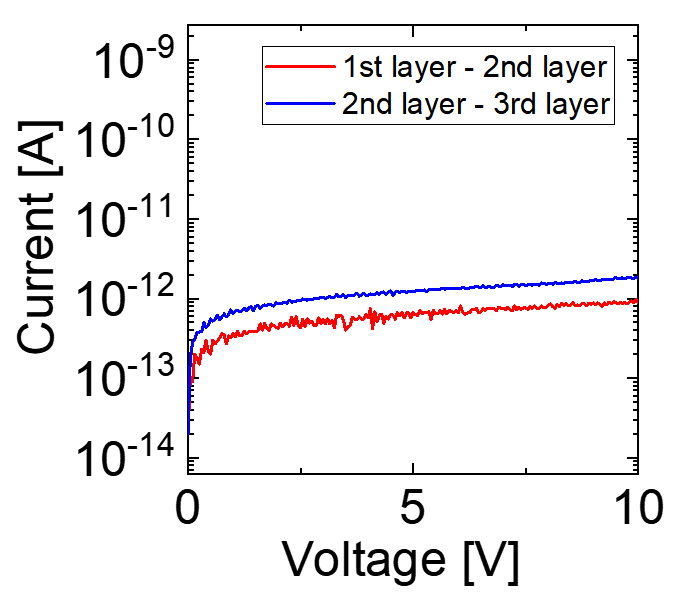


**Figure S3 | Experimental verification of inter-layer leakage suppression.**

Measured current between vertically adjacent bit lines under read bias, showing negligible leakage (~pA level) through grounded neighboring lines, confirming sufficient isolation by the 100-nm-thick SiO_2_ layer.

**
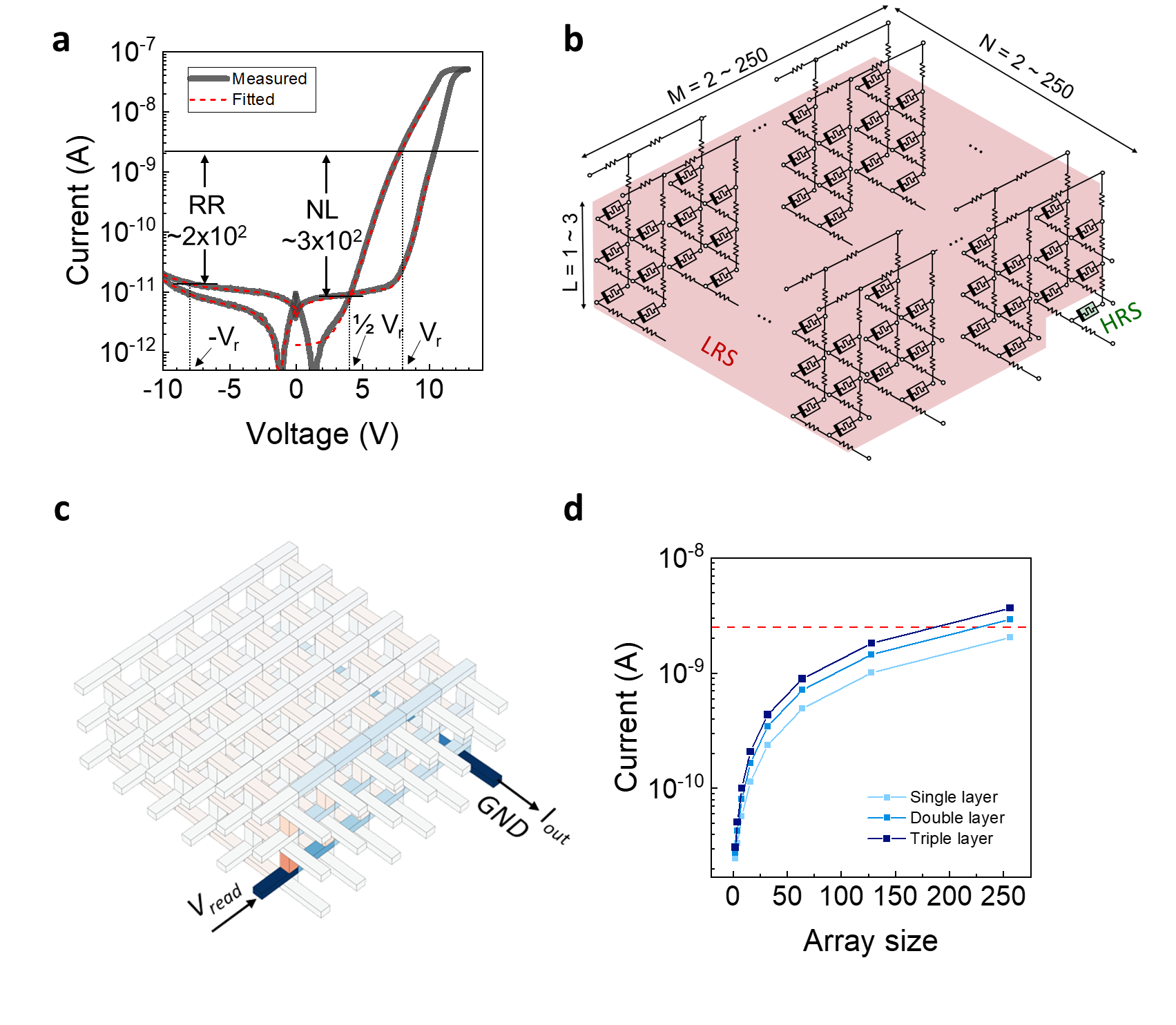
**

**Figure S4 | Simulation and modeling of sneak current behavior in PTHT V-RRAM arrays.**
(a) Measured and fitted I-V curves of the PTHT memristor used for simulation, showing ~2×10^2^ rectification ratio and ~3×10^2^ nonlinearity. (b) Equivalent circuit of the modeled 3-layer V-RRAM array, where only the selected cell is in HRS and all others are in LRS, representing the worst-case scenario. (c) Simulated sneak current distribution map of a 6×6×3 V-RRAM array. Deep blue represents forward currents and red represents reverse currents, with color intensity indicating current magnitude. The weak color intensity of the sneak path demonstrates that undesired sneak currents are effectively suppressed, and the majority of current flow remains confined to the intended read path. (d) Simulated read current of the selected HRS cell as a function of array size (N = M) for 1-, 2-, and 3-layer arrays under the worst-case configuration. The dashed red line indicates the read current of an LRS cell. As the number of layers increases, the total read current increases due to additional possible sneak paths. However, HRS and LRS currents remain distinguishable up to approximately N = 190, confirming stable scalability in multilayer arrays.


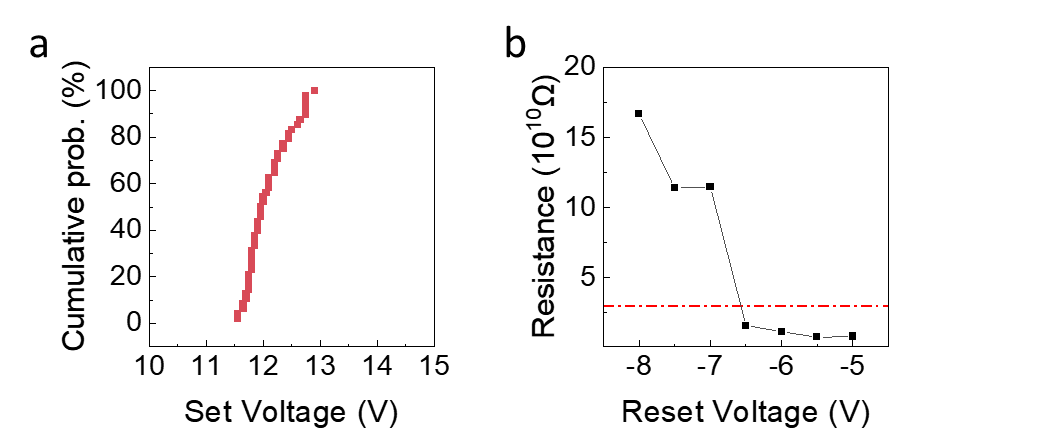


**Figure S5 | Set and reset voltage analysis**

(a) Set voltage distribution of 48 devices within a single V-RRAM array. (b) The resistance state of the V-RRAM PTHT device depending on the applied reset voltage, where an abrupt reset is observed around –7 V.


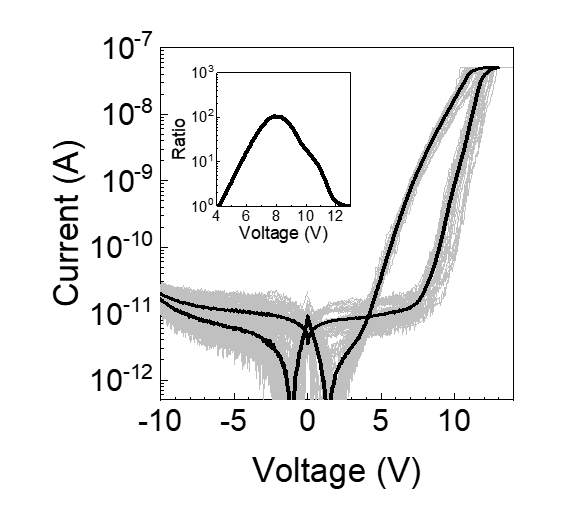


**Figure S6 | I-V curves of 48 devices within a single V-RRAM array (gray), together with the averaged I-V curve (black).**

The inset shows the LRS/HRS ratio calculated from the averaged I-V curve at each read voltage, with the maximum ratio observed at 8 V.


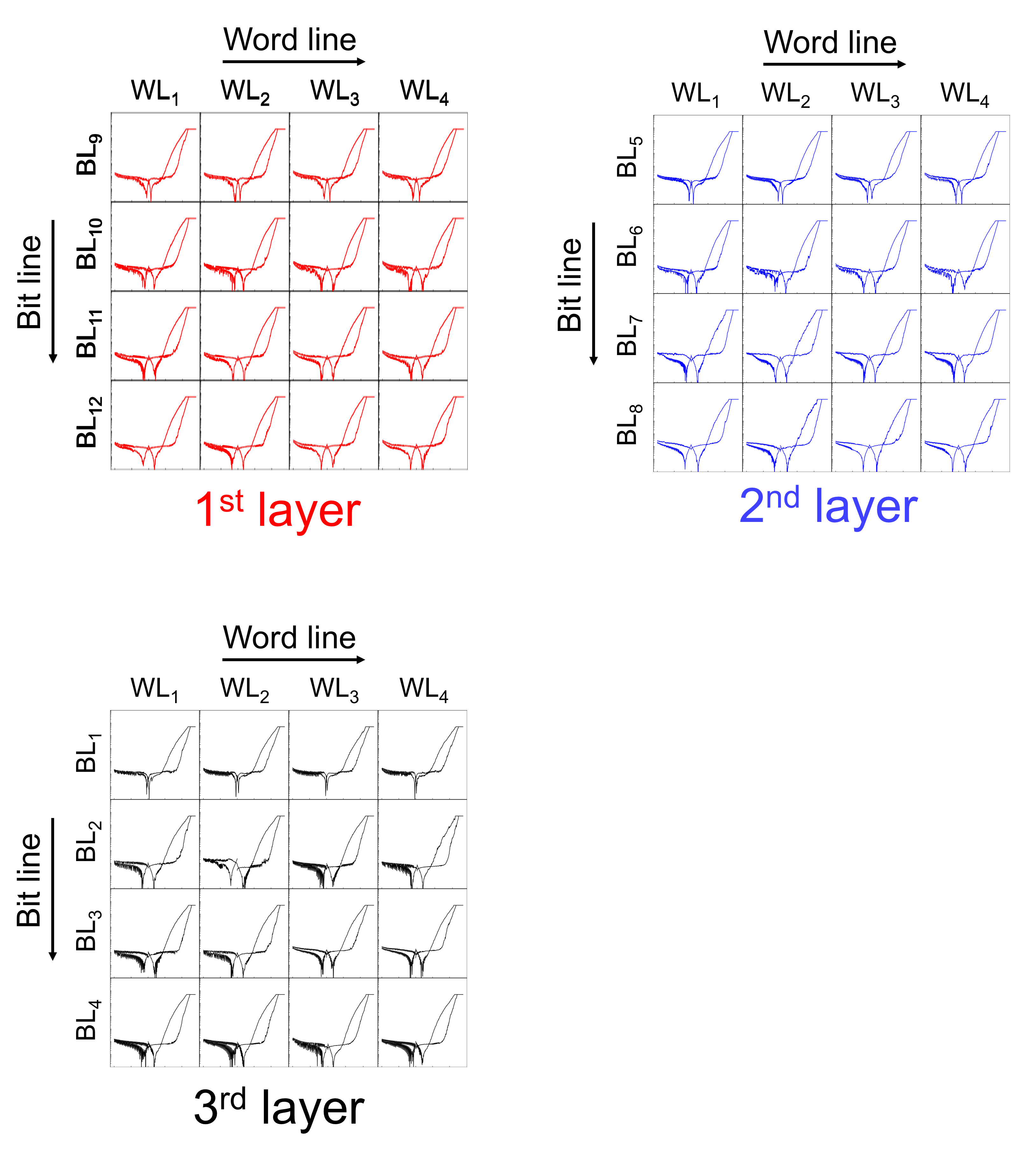


**Figure S7 | I-V curves from devices at various positions within the 3-layer V-RRAM array, demonstrating uniform electrical characteristics across the array.**


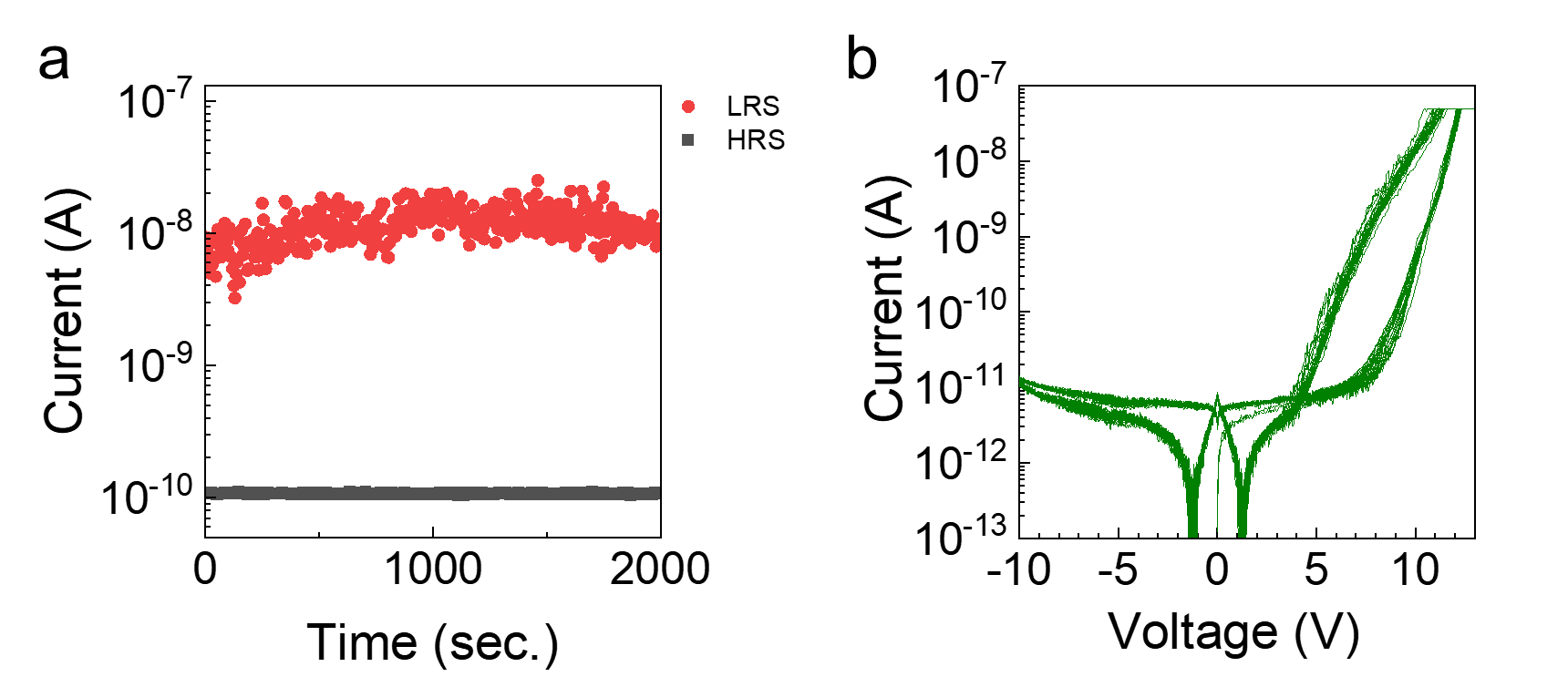


**Figure S8 | Reliability characteristics of the PTHT V-RRAM device.**

(a) Retention data measured at a read voltage of 8 V. (b) I-V curves for DC cycling over 30 cycles.


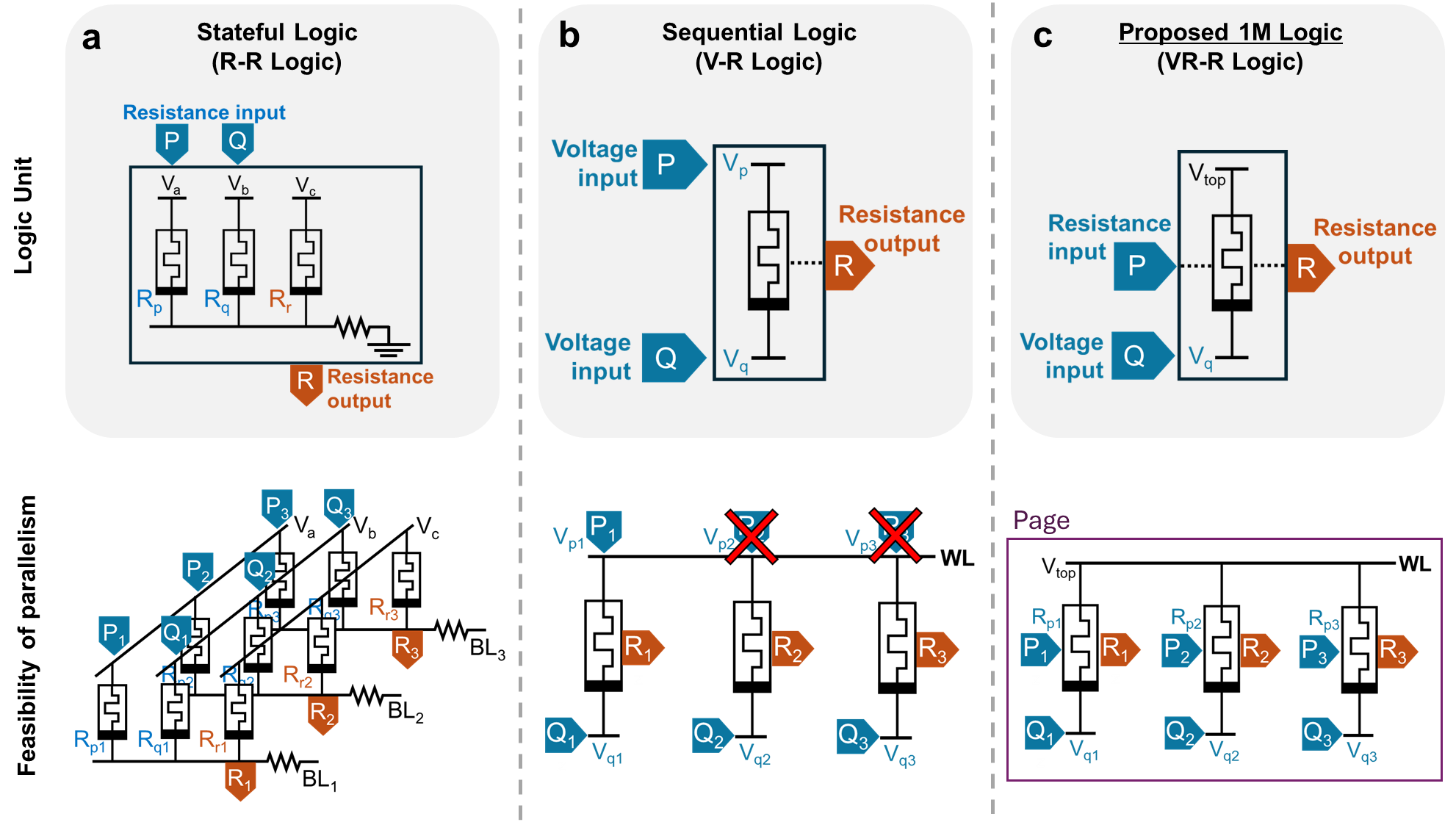


**Figure S9 | Comparison of memristor-based logic-in-memory schemes.**

(a) stateful logic (R-R), (b) sequential logic (V-R), and (c) proposed 1M logic (VR-R). Detailed descriptions of each scheme are provided in Supplementary Note S2.


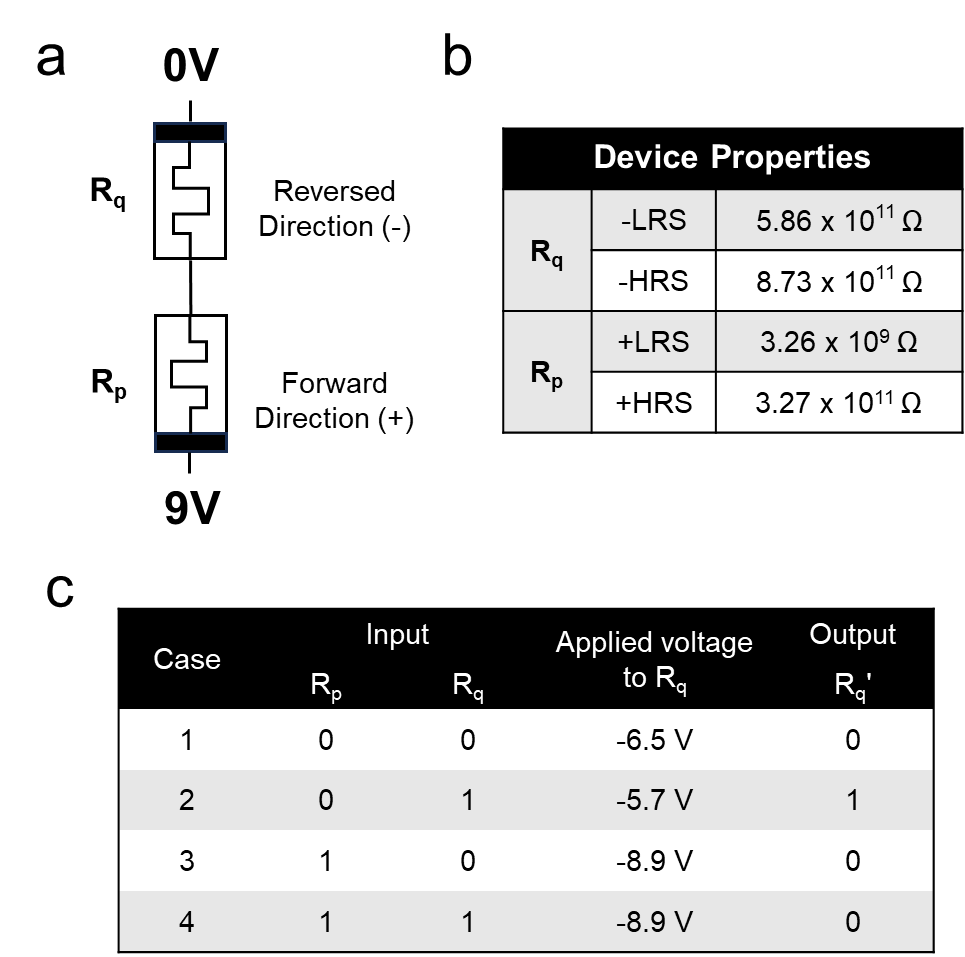


**Figure S10 | Details of the 2M logic operation.**

(a) Anti-serial configuration of the 2M logic unit. (b) Resistance of the V-RRAM devices depending on orientation and state. (c) The voltage applied to R_q_ for each case during the 2M logic operation, determined by voltage division. Consequently, R_q_ retains the LRS only in Case 2, consistent with the RNIMP truth table.


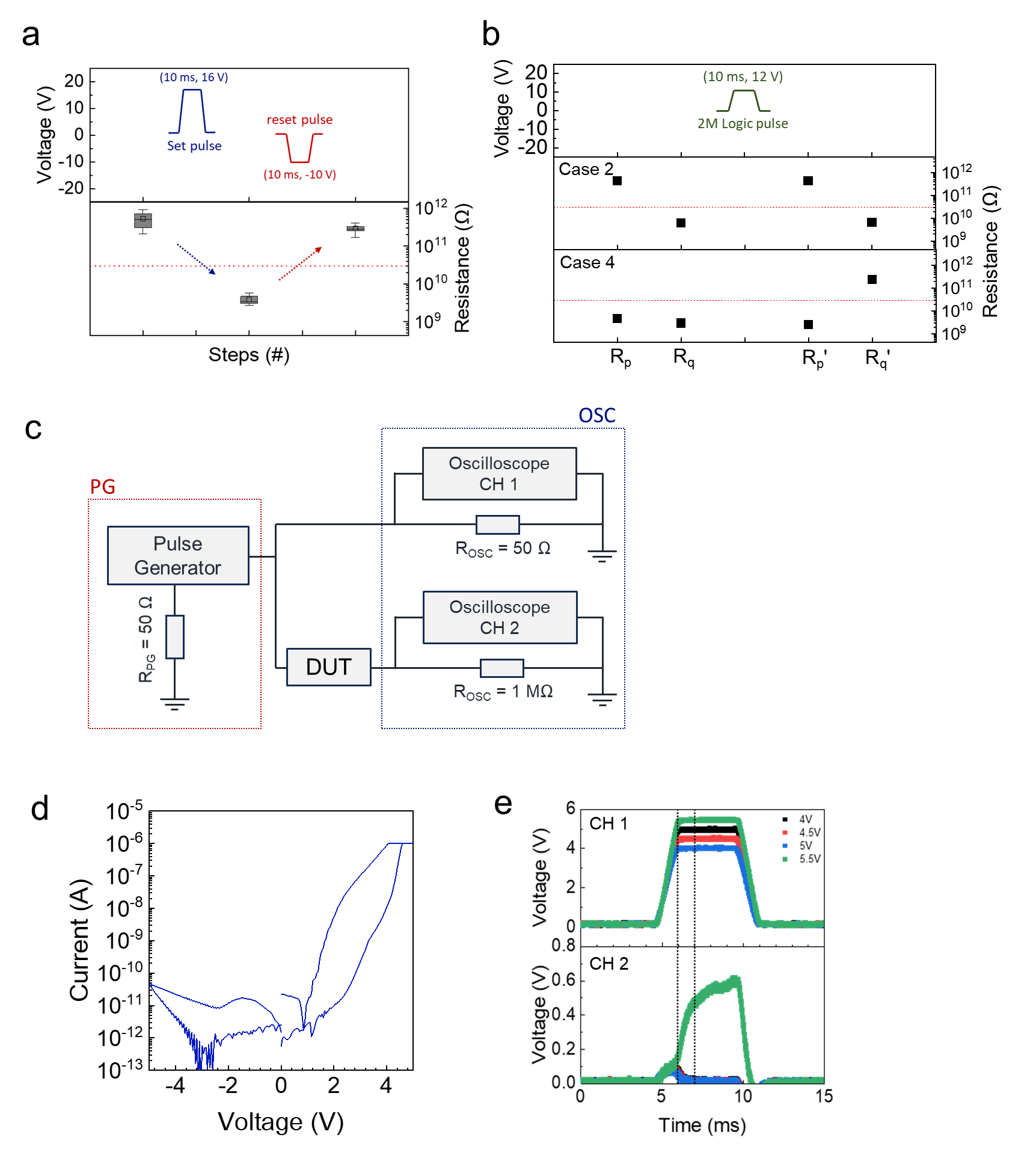


**Figure S11 | Pulsed voltage switching and logic operation.**

(a) **Set/Reset switching under voltage pulses** (10 ms, +16 V for set; 10 ms, −10 V for reset). (b) **2M logic under a single pulsed bias** (10 ms, 12 V). In the anti-serial configuration, voltage division preserves **R­_q_** in LRS for **Case 2**, whereas **Case 4** drives **R_q_** above the reset threshold and switches it to HRS. (c) **Pulse-measurement setup schematic.** Pulse generator drives the DUT. Oscilloscope monitors CH1 and CH2. (d) I-V characteristics of the vertical Pt/HfO_2_ (4 nm)/TiN (PHT) device. (d) **Transient set switching in the PHT device,** measured using the pulse-measurement setup. CH1/CH2 waveforms under 5 ms pulses (5.5 V) show a sharp CH2 rise within ~1 ms, indicating the set transition.


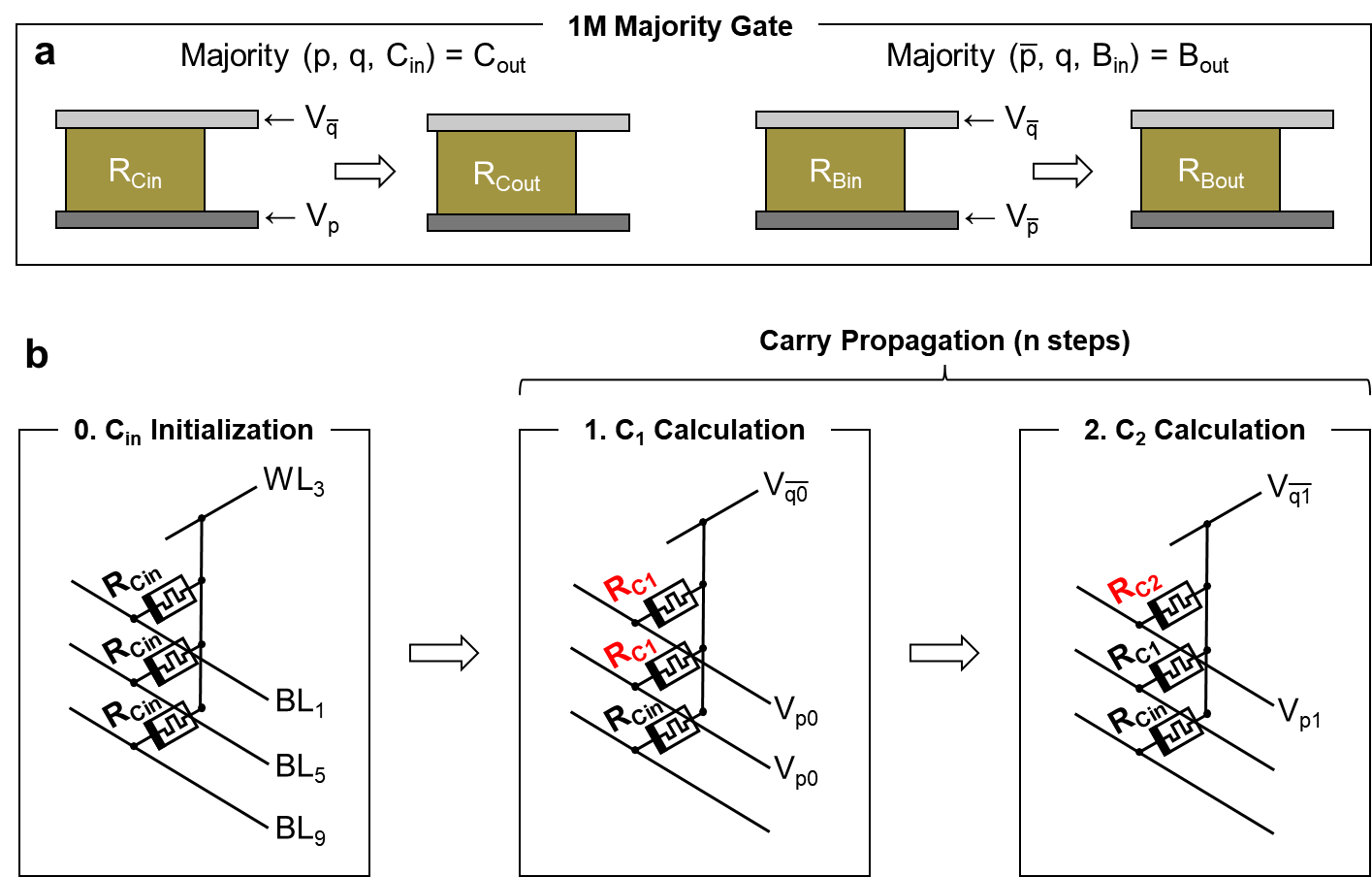


**Figure S12 | 1M majority gate and carry propagation process.**

(a) The majority gate can be utilized for both addition and subtraction operations. Carry-out (*C_out_*) is obtained using a majority gate of carry-in (*C_in_*), input p and input q. In contrast, borrow-out (*B_out_*) is derived from a majority gate of borrow-in (*B_in_*), the complement of input p (p̅) and input q. In both cases, the voltage applied to the top electrode for carry and borrow remains the same as q̅. In contrast, the voltage applied to the bottom electrode differs, being input p for carry and p̅ for borrow operations. The logic operation is executed based on the pre-programmed resistance states of the memristors, which represent the carry-in or borrow-in inputs. (b) Schematic diagram illustrating the carry propagation process.

**
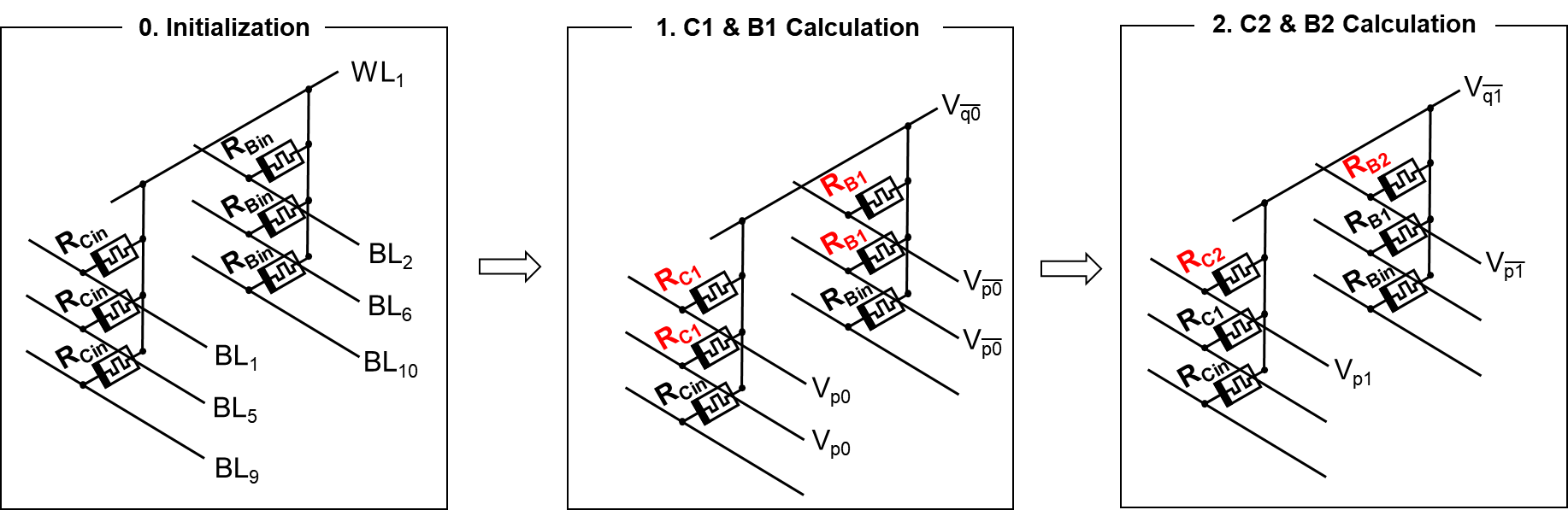
**

**Figure S13 | Schematic diagram illustrating the parallel execution of carry and borrow propagation.**


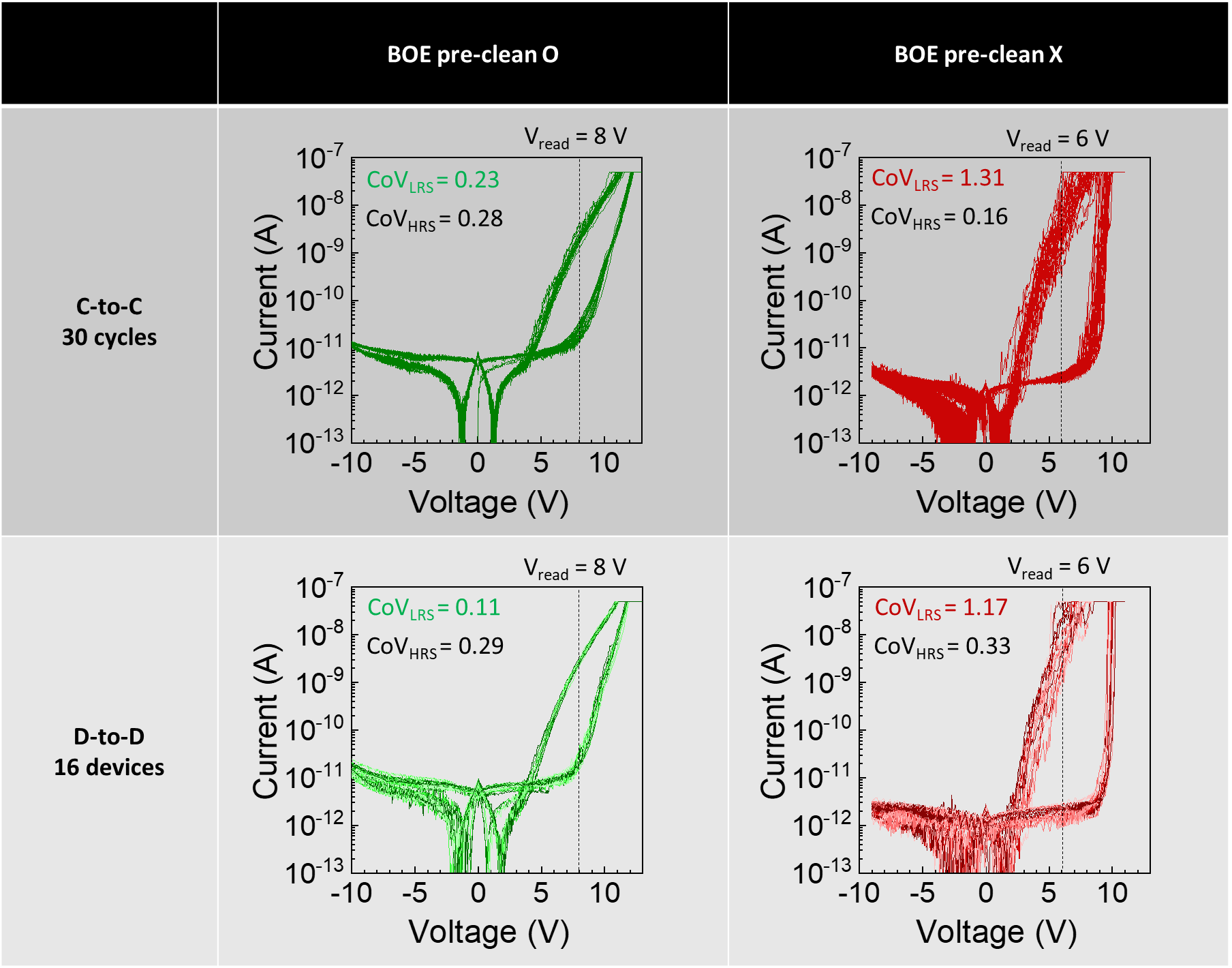
**Figure S14 | Impact of BOE pre-clean on variability.**

Cycle-to-cycle and device-to-device I-V characteristics **with** (left, buffered oxide etchant (BOE) pre-clean “O”) and **without** (right, BOE pre-clean “X”) the BOE pre-clean after hole etching and O_2_ plasma ashing.

Buffered oxide etchant (BOE) pre-clean was introduced to remove TiON before the dielectric film deposition. This step leads to a more uniform trap distribution in Al:HfO_2_ and promotes gradual switching, improving reproducibility across cycles and devices.


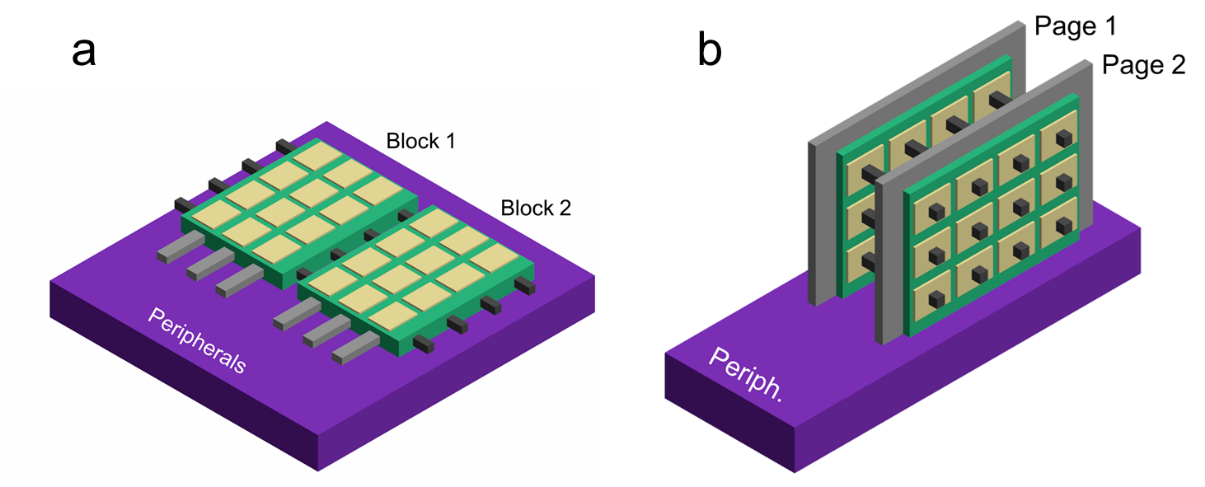


**Figure S15 | Schematic comparison of 2D and 3D V-RRAM arrays.**

(a) schematic of a conventional 2D planar crossbar RRAM array, where logic operations are performed line-wise with individual word lines accessing a single horizontal row. (b) Schematic of the proposed 3D vertical RRAM (V-RRAM) array, in which a single word line services an entire vertical page, enabling page-wise logic operations across stacked cells.


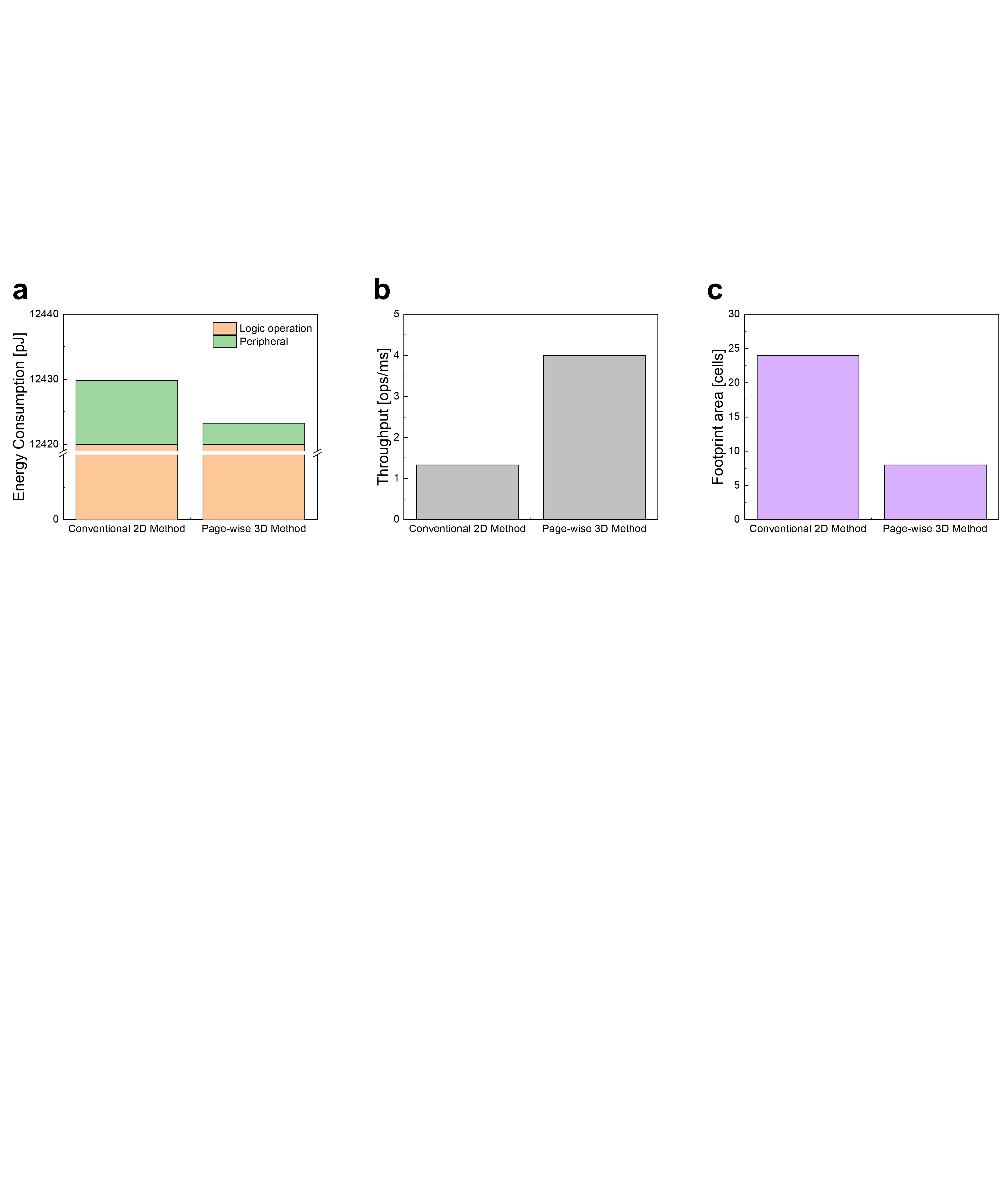


**Figure S16 | Performance benchmarking of 2D vs. 3D XOR operations.**

Comparison of energy consumption (a), throughput (b), and footprint area (c) for a 12-bit XOR operation between the conventional 2D planar method and the proposed V-RRAM page-wise method. The page-wise method achieves nearly identical energy efficiency while improving throughput and area efficiency by 3× respectively.

**Table S1 | Comparison of Logic-in-Memory Architectures**

**
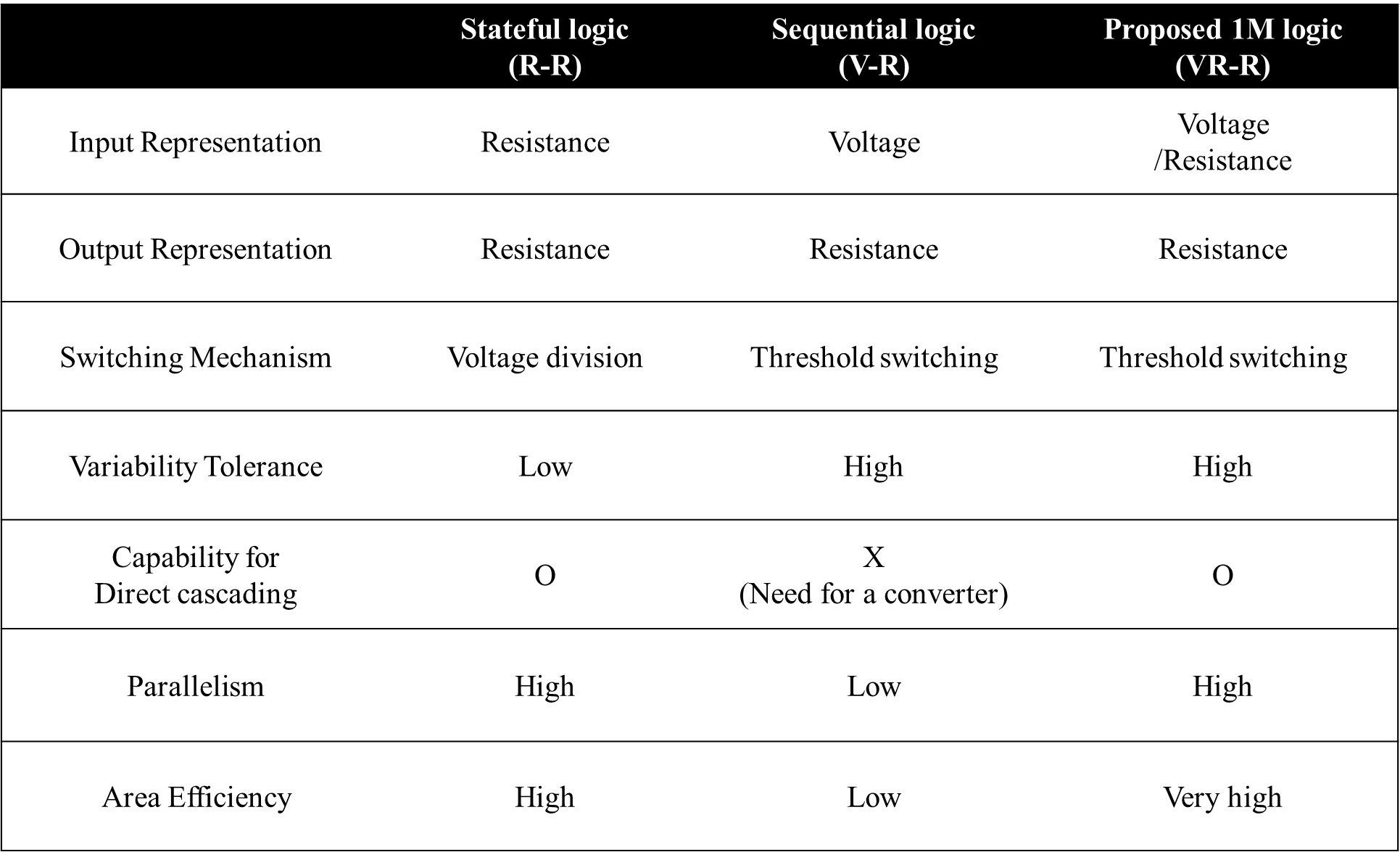
**

**Table S2 | Truth** **table of the majority gate.**

**
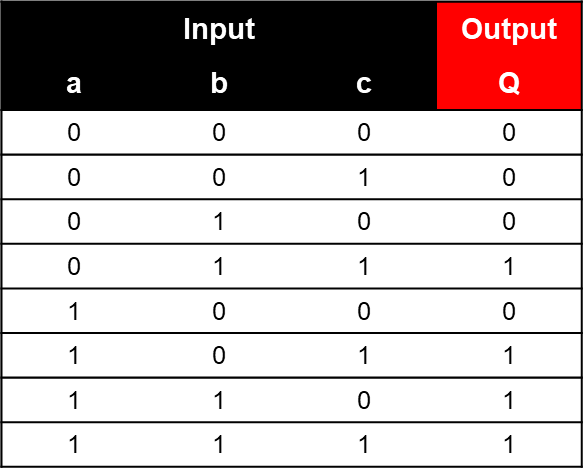
**

The majority gate is a logic gate that outputs 1 when at least two of the three inputs are 1, which can be utilized to generate carry-out during addition and borrow-out during subtraction operations.

**Table S3 | Logic table when p, q, and *C_in_* (or *B_in_*) are provided as inputs to a majority 1M logic gate.**


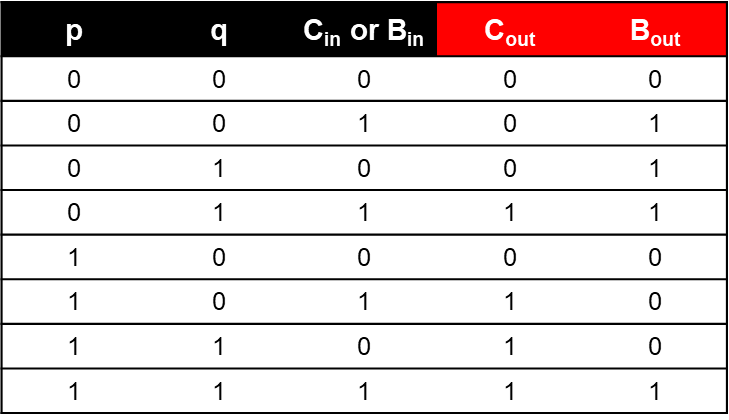


When *C_in_* is the input, the output is *C_out_*. And when *B_in_* is the input, the output is *B_out_*.

**Table S4 | Energy consumption associated with individual logic operations (AND, OR, and RNIMP gates) and peripheral circuitry (MUX and word-line driving).**

|  | AND gate | OR gate | RNIMP gate | MUX + Word line driving |
| --- | --- | --- | --- | --- |
| Energy consumption | 0.1 pJ/bit | 1035 pJ/bit | 0.15 pJ/bit | 1.1 pJ/line |

Values are reported per bit for logic switching events and per line for word-line driving.

**Supplementary Note S1**

**Sneak Path Definition, Measurement, and Simulation Analysis of PTHT V-RRAM**

**Supplementary** **Figure S2** schematically illustrates the sneak current behavior in the proposed V-RRAM structure.

In **Figure S2a**, the intended read current flows from the selected word line (WL) through the target cell to the selected bit line (BL). However, unintended paths can form when current passes through unselected cells, floating BLs, and intermediate WLs, eventually reaching the selected BL. Although such paths in V-RRAM may cross multiple layers and pages, they are primarily composed of two forward-biased and one reverse-biased cell, structurally identical to those in conventional 2D crossbar arrays. Therefore, the core sneak-path topology remains unchanged, with vertical stacking being the only structural difference.

Besides these paths, **Figure S2b** shows the potential for additional vertical leakage current paths in hole-type V-RRAM structures. Since the resistive switching layers are stacked vertically along the sidewalls, insufficient isolation between neighboring BLs can allow leakage current to pass through the inter-layer isolation layer, possibly linking adjacent memory layers. This vertical leakage current creates extra sneak channels not present in planar 2D arrays.

To experimentally verify the effect of such inter-layer coupling, a bias voltage was applied to a specific BL while grounding the adjacent BL above or below it. As shown in **Figure S3**, the measured inter-layer leakage current under read bias was around picoamperes, confirming that the 100-nm-thick SiO_2_ isolation layer effectively reduces vertical leakage.

The self-rectifying and nonlinear characteristics of the PTHT memristor, which block reverse-biased sneak currents, have been experimentally confirmed in earlier work^[S1]^. Even in the worst-case scenario (all unselected cells in the low-resistance state, LRS), the high-resistance state (HRS) cell could be reliably read without interference in a 4×4, two-layer array.

To further assess scalability, circuit-level simulations were conducted using the experimentally fitted PTHT I-V characteristics and a measured line resistance (TiN, 50 nm, 38 Ω/sq). **Figure S4a** displays the measured and fitted I-V curves, while **Figure S4b** illustrates the equivalent circuit used in HSPICE simulation under the worst-case condition.

For a practical 6×6×3 array comparable to the experimental scale, the simulated current-distribution map (**Figure S4c**) shows that most HRS read current is confined to the intended path, indicating effective suppression of undesired sneak currents. Scalability was then examined by varying the array dimension (N = M = 2–250) while fixing the number of layers (L = 3). **Figure S4d** illustrates the simulated read current of the selected HRS cell as a function of N, where the dashed red line denotes the LRS read current. As the number of layers increases, the sneak current increases due to additional possible sneak paths. However, HRS and LRS currents remain distinguishable up to approximately N = 190, beyond which overlap occurs, marking the onset of read-margin collapse. These results collectively verify that the PTHT V-RRAM structure effectively suppresses both lateral and vertical sneak paths, ensuring read integrity and scalability even in high-density three-layer configurations.

**Supplementary Note S2**

**Comparison of Logic-in-Memory Architectures**

Memristor-based logic-in-memory (LIM) architectures have traditionally developed along two main directions: stateful logic (R-R logic) and sequential logic (V-R logic). Both methods utilize the resistive switching properties of memristors to perform Boolean logic operations directly within memory arrays. Although they have contributed to the foundation of LIM research, each approach faces inherent limitations that have restricted large-scale adoption. **Figure S9** illustrates the operational principles, and **Table S1** outlines the advantages and weaknesses of these two traditional logic families, contrasting them with the proposed VR-R logic that aims to overcome their shortcomings.

***Stateful Logic (R-R Logic)***

In stateful logic, both inputs and the output are encoded as memristor resistance states (**Figure S9a**). This uniform representation allows for direct cascading, as the resistance output of one logic operation can be used as the input for subsequent steps. In principle, stateful logic also enables highly parallel operation. Because identical voltages are applied to devices along the same word line, operations can be performed in parallel across multiple bit lines, making the level of parallelism scale with the number of bit lines, as shown in the lower panel of **Figure S9a**. However, this approach depends on resistive voltage division, which makes it highly sensitive to variations between devices. Even small deviations in resistance values, especially in the high-resistance state, can distort the voltage distribution and cause unintended switching. These effects can build up during cascaded operations, leading to unstable behavior. As a result, demonstrations of stateful logic have so far been limited to a few operation steps or simulations.

***Sequential Logic (V-R Logic)***

Sequential logic encodes inputs as applied voltages, while the output is stored as the final resistance state of the device. This architecture demonstrates robustness against device variability because switching occurs whenever the applied voltage exceeds the intrinsic threshold, regardless of resistive mismatch. However, sequential logic faces two structural limitations. First, the mismatch between voltage-based inputs and resistance-based outputs prevents direct cascading without additional logic state conversion circuitry. Second, since each device needs an independent voltage input, efficient parallel operation cannot be achieved in an array layout. This limitation is clearly shown in **Figure S9b** (lower panel), where only one device can be addressed at a time, and attempts to operate multiple devices simultaneously fail due to the need for separate bias lines. As a result, sequential logic is inherently unsuitable for efficient large-scale parallelism. Efforts to extend parallelism by selecting diagonal cells in crossbar arrays have consistently led to significant area inefficiency, further limiting scalability.

***Proposed 1M Logic (VR-R Logic).***

The proposed 1M logic combines the strengths of both stateful and sequential paradigms while eliminating their inherent weaknesses. In this approach, one input is encoded as a resistance state, and the other as a bottom-electrode voltage bias. Simultaneously, the top electrode is held at a fixed, predetermined operating voltage (**Figure S9c**). This setup ensures reliable and reproducible switching, as operation is triggered by a direct voltage above the threshold rather than unreliable resistive division. Additionally, since both the input and output are stored as resistance states, seamless cascading capability remains intact. Because only one device is needed to perform a 1-bit operation, the number of devices required for multi-bit operations is significantly reduced compared to stateful logic, as shown in the lower panel of **Figure S9**. Moreover, leveraging these advantages, the proposed operation can be applied to 3D vertical RRAM arrays, where devices along the same word line can be biased simultaneously, enabling page-wise parallel logic execution across entire vertical stacks. The 1M logic thus provides both a technical solution to long-standing issues of variability and cascading, as well as a conceptual advance toward scalable arithmetic and complex in-memory computing within memristor arrays. **Table S1** offers a comparison of the characteristics of each logic method.

**Supplementary Note S3**

**Rationale for V_op_ = 9 V in the 2M Logic unit: Voltage-Division in Anti-Serial Self-Rectifying Memristors.**

The operation voltage of 9 V for the 2M logic unit can be explained by analyzing the voltage division mechanism between the two anti-serially connected self-rectifying memristors (**Figure S10**). During the logic operation, where voltage division occurs, the reverse-biased memristor (R_q_) exhibits a resistance comparable to the HRS (~5.86–8.73 × 10^11^ Ω), irrespective of its programmed state, owing to the self-rectifying characteristics of the device. Meanwhile, under the applied operation voltage, the forward-biased device (R_p_) exhibits resistance values that differ by approximately two orders of magnitude depending on whether it has been programmed into the LRS or HRS (~3.26 x 10^9^ Ω or ~3.27 x 10^11^ Ω).

As a result, when R_p_ is in HRS (Cases 1 and 2), the applied voltage is distributed more evenly between R_p_ and R_q_, and the voltage drop across R_q_ is below the reset threshold (~7 V), thereby preserving its resistance state. By contrast, when R_p_ is in LRS (Cases 3 and 4), most of the applied voltage is dropped across R_q_, and this voltage (~8.9 V) exceeds the reset threshold (~7 V), thereby inducing the reset of R_q_. Accordingly, when R_q_ is initially in the LRS (Cases 2 and 4), it remains preserved in Case 2 but is reset to HRS in Case 4, thereby establishing the RNIMP operation. Based on this voltage division mechanism, the operation voltage was determined to be 9 V.

**Supplementary Note S4**

**Pulse-Induced Switching in Self-Rectifying V-RRAM**

Pulse-induced set and reset switchings of the V-RRAM PTHT device were confirmed, as shown in **Figure S11a**. The pulses were applied using a pulse generator (PG, Agilent 81110 A), and the conductance was read with a semiconductor parameter analyzer (SPA, HP 4155A). Because the PTHT device is too insulating and exhibits a noticeable capacitive component, the initial pulse produces a charging peak (~2 ms) that obscures the set transition. In addition, both the LRS and HRS are substantially larger than the 1 MΩ feedback resistor of the oscilloscope (OSC), making the voltage across the device under test (DUT) difficult to distinguish from measurement noise. Accordingly, DC readout with the SPA was employed to obtain the results in **Figure S11a** and **b** instead of using OSC. Under the same method (PG-driven pulse followed by DC readout), **Figure S11b** confirms that, in the 2M logic, the voltage division across the anti-serial configuration **preserves R_q_ in the LRS for Case 2**. In contrast, for **Case 4, it drives R_q_ above the reset threshold, switching it to the HRS**. This behavior is consistent with the manuscript in **Figure 4d**.

To verify that the self-rectifying V-RRAM device indeed switches under pulsed excitation, a Pt/HfO_2_/TiN (PHT) device with the same vertical architecture but a thinner HfO_2_ layer (4 nm), which exhibits a higher operating current at a given bias, was evaluated. The measurement setup, comprising a PG and OSC is shown in **Figure S11c**. The I-V characteristic of the PHT device is shown in **Figure S11d**. In this configuration, a set transition was directly observed: within ~1 ms, the DUT resistance decreased, and the oscilloscope-measured voltage rose sharply, as shown in **Figure S11e**. This result **confirms pulse-induced switching in the vertical V-RRAM platform**.

**Supplementary Note S5**

**Energy Efficiency, Throughput and Area Efficiency Benchmarking of 2D vs. 3D Memristor Logic**

To quantitatively address energy efficiency, the energy cost per Boolean operation was evaluated, incorporating both device-level switching and peripheral overhead (see **Table S4** and **Figure S15**). For a representative 12-bit XOR, the conventional 2D array required 9 sequential steps and 24 cells with a total energy of ~12,430 pJ, corresponding to a throughput of 1.33 ops/ms. In contrast, the proposed page-wise scheme completed the same operation in 3 steps and 8 cells with ~12,423 pJ, yielding a throughput of 4 ops/ms (see **Figure S16** for direct comparison). Thus, although the net energy reduction was minimal (~0.05%) under present device conditions, the throughput and the area efficiency increased by ~3×. Significantly, when adopting lower power switching devices reported in recent literature (<2.5 pJ/bit), peripheral driving dominates the energy budget. Under these conditions, the page-wise scheme reduced energy per operation by ~13.9 % compared to the 2D approach. These results highlight that while the immediate benefit of the proposed approach is throughput and area efficiency, its energy advantage will become increasingly significant as device technology advances.

**Supplementary Note S6**

**Implementation of carry and borrow propagation using 1M logic-based Majority gate in V-RRAM**

Carry and borrow propagation in V-RRAM-based ALUs were implemented using a majority gate based on 1M logic. As shown in **Table S2**, a majority gate is defined as a logic gate that outputs '1' when at least two out of the three inputs are '1', and outputs '0' otherwise.

As illustrated in **Table S3**, employing the majority gate enables the propagation of carry during the addition of p and q, and the propagation of borrow during the subtraction of p and q. As depicted in **Figure S12a**, the majority gate can be implemented in a single step using 1M logic. The *C_in_* is stored in the memristor resistance state, while the top electrode is applied with the voltage corresponding to input $\bar{q}$, and the bottom electrode is applied with the voltage of input p. The majority gate operation consequently updates the memristor resistance state to represent the *C_out_* of the next bit. In this configuration, a resistance logic '1' corresponds to the low-resistance state (LRS), and a voltage logic '1' corresponds to ground, consistent with the convention used in AND and OR 1M logic.

For borrow propagation, the *B_in_* is initially stored in the memristor. By applying a voltage corresponding to input $\bar{q}$ to the top electrode and input $\bar{p}$ to the bottom electrode, the final memristor resistance state after the operation corresponds to the *B_out_*.

**Figure S12b** shows the procedure for carry propagation using this logic scheme:

- **Step 0:** Initialize the resistance states of the three vertically stacked V-RRAM devices to represent the input *C_in_*
- **Step 1:** Apply the voltage corresponding to input $\bar{q_{0}}$ to the top electrode, while applying the voltage corresponding to input p_0_ to the bottom electrodes of the second and third layers. This voltage configuration causes resistive switching in these layers, thereby updating the intermediate logic state *C_1_*.
- **Step 2:** Apply the voltage corresponding to input $\bar{q_{1}}$ to the top electrode and that of input p_1_ only to the bottom electrode of the third layer. This selectively modulates the resistance state of the third-layer device, resulting in the final logic state *C_2._*

Using this sequential voltage application, n-bit carry propagation can be completed within n steps without any additional data movement. As shown in **Figure S12a**, borrow propagation utilizes the same majority gate configuration with the same top electrode voltage while inverting the bottom electrode voltage to input $\bar{p}$. This enables parallel propagation of carry and borrow across devices sharing the top electrode, allowing multiple ALUs to perform carry and borrow propagation simultaneously within n steps, as demonstrated in **Figure S13**.

**Supplementary References**

[S1] S. S. Kim, S. K. Yong, J. Kim, J. M. Choi, T. W. Park, H. Y. Kim, H. J. Kim, C. S. Hwang, Fabrication of a Hole-Type Vertical Resistive-Switching Random-Access Array and Intercell Interference Induced by Lateral Charge Spreading, *Adv. Electron. Mater.* **2023**, *9*
